# Multimodal genome-wide survey of progressing and non-progressing breast ductal carcinoma in-situ

**DOI:** 10.1186/s13058-024-01927-1

**Published:** 2024-12-04

**Authors:** Marija Debeljak, Soonweng Cho, Bradley M. Downs, Michael Considine, Brittany Avin-McKelvey, Yongchun Wang, Phillip N. Perez, William E. Grizzle, Katherine A. Hoadley, Charles F. Lynch, Brenda Y. Hernandez, Paul J. van Diest, Wendy Cozen, Ann S. Hamilton, Debra Hawes, Edward Gabrielson, Ashley Cimino-Mathews, Liliana D. Florea, Leslie Cope, Christopher B. Umbricht

**Affiliations:** 1 Department of Surgery, Johns Hopkins University School of Medicine, Baltimore, MD USA; 2 Institute for Nanobiotechnology, Johns Hopkins University, Baltimore, MD USA; 3 Department of Oncology, Johns Hopkins University School of Medicine, Baltimore, MD USA; 4 Department of Pathology, Johns Hopkins University School of Medicine, Baltimore, MD USA; 5 Department of Biostatistics, Johns Hopkins University School of Medicine, Baltimore, MD USA; 6 Department of Pathology, University of Alabama at Birmingham School of Medicine, Birmingham, AL USA; 7 Department of Genetics, Lineberger Comprehensive Cancer Center, The University of North Carolina at Chapel Hill, Chapel Hill, NC USA; 8 Department of Epidemiology, College of Public Health, University of Iowa, Iowa City, IA USA; 9 Population Sciences in the Pacific-Program, University of Hawaii Cancer Research Center, Honolulu, HI USA; 10 Department of Pathology, University Medical Center Utrecht, Utrecht, The Netherlands; 11 Department of Medicine, School of Medicine, Susan and Henry Samueli College of Health Sciences, University of California at Irvine, Irvine, CA USA; 12 Department of Population and Public Health Sciences, Keck School of Medicine, University of Southern California, Los Angeles, CA USA; 13 Department of Pathology and Laboratory Medicine, Keck School of Medicine, Children’s Hospital Los Angeles, University of Southern California, Los Angeles, CA USA; 14 Department of Genetic Medicine, Johns Hopkins University School of Medicine, Baltimore, MD USA; 15 The Johns Hopkins University School of Medicine, Ross Building, Room 743, 720 Rutland Ave, Baltimore, MD 21205 USA

**Keywords:** DCIS progression, Genome-wide survey, Transcriptome, Methylome, DNA copy number variation, Alternative splicing, Gene set enrichment analysis

## Abstract

**Background:**

Ductal carcinoma in-situ (DCIS) is a pre-invasive form of invasive breast cancer (IBC). Due to improved breast cancer screening, it now accounts for ~ 25% of all breast cancers. While the treatment success rates are over 90%, this comes at the cost of considerable morbidity, considering that the majority of DCIS never become invasive and our understanding of the molecular changes occurring in DCIS that predispose to invasive disease is limited. The aim of this study is to characterize molecular changes that occur in DCIS, with the goal of improving DCIS risk stratification.

**Methods:**

We identified and obtained a total of 197 breast tissue samples from 5 institutions (93 DCIS progressors, 93 DCIS non-progressors, and 11 adjacent normal breast tissues) that had at least 10-year follow-up. We isolated DNA and RNA from archival tissue blocks and characterized genome-wide mRNA expression, DNA methylation, DNA copy number variation, and RNA splicing variation.

**Results:**

We obtained all four genomic data sets in 122 of the 197 samples. Our intrinsic expression subtype-stratified analyses identified multiple molecular differences both between DCIS subtypes and between DCIS and IBC. While there was heterogeneity in molecular signatures and outcomes within intrinsic subtypes, several gene sets that differed significantly between progressing and non-progressing DCIS were identified by Gene Set Enrichment Analysis.

**Conclusion:**

DCIS is a molecularly highly heterogenous disease with variable outcomes, and the molecular events determining DCIS disease progression remain poorly defined. Our genome-wide multi-omic survey documents DCIS-associated alterations and reveals molecular heterogeneity within the intrinsic DCIS subtypes. Further studies investigating intrinsic subtype-stratified characteristics and molecular signatures are needed to determine if these may be exploitable for risk assessment and mitigation of DCIS progression. The highly significant associations of specific gene sets with IBC progression revealed by our Gene Set Enrichment Analysis may lend themselves to the development of a prognostic molecular score, to be validated on independent DCIS cohorts.

**Supplementary Information:**

The online version contains supplementary material available at 10.1186/s13058-024-01927-1.

## Background

Ductal carcinoma in situ (DCIS) is a non-obligate precursor of invasive breast cancer (IBC), characterized by abnormal ductal epithelial cells that have not invaded through the ductal basement membrane and are therefore considered noninvasive. Widespread mammographic screening has resulted in the dramatic increase in diagnosis of DCIS [1, 2], which currently accounts for up to 25% of newly diagnosed breast cancer in the US [3, 4]. Treatment for this pre-invasive lesion varies widely and ranges from surveillance in low grade cases to total mastectomy with or without adjuvant radiation (RT) and/or endocrine therapy (ET) In higher grade cases. While the success rate of optimal treatment protocols for DCIS is over 90%, this comes at the cost of significant morbidity [5]. Considering that the majority of DCIS never become invasive carcinomas [6–9], less aggressive treatment protocols would spare patients at low risk for developing invasive cancer from significant morbidity [10].

The molecular events determining disease progression remain poorly defined. Breast cancer-specific markers may not be characteristic of the early events determining which DCIS is destined to progress. Even in studies focusing specifically on DCIS, most of the data are derived from samples that harbor synchronous invasive cancer [5, 11, 12], and are therefore unlikely to be fully representative of DCIS in patients without invasive cancer [13].

Several decades of investigations have identified a set of clinical and pathological variables that have prognostic value, such as age, extent of disease, estrogen receptor (ER) status, nuclear grade, and surgical resection margins, but these have proven insufficient to allow meaningful treatment stratification [5, 14, 15], and 75% of women receive RT without clear guidance on who can safely avoid it [4].

Molecular characterizations of DCIS have led to the development of biomarker panels that improve risk assessment. The Oncotype DX^®^ DCIS Score (EXACT Sciences, Madison, WI, USA) is a 12-gene assay that estimates 10-year in-breast recurrence risk (IBR) in women identified as having low risk DCIS treated with breast-conserving surgery alone [16, 17]. However, given the narrow selection criteria of these trials, the Oncotype DX DCIS Score is currently only applicable to post-surgery DCIS patients at low risk for recurrence, which has limited its clinical use so far. Another currently available assay, DCISionRT^®^ (PreludeDx, Laguna Hills, CA, USA), uses a recurrence risk score based on a combination of clinical and pathologic factors. The initial studies were performed retrospectively on DCIS patients treated with breast conserving surgery ± RT and were shown to be prognostic for risk of recurrence and predictive for RT-benefit [18]. Importantly, neither assay has been tested in prospective, randomized clinical trials and their role in clinical management of DCIS remains to be determined.

In this study, we identified a large multi-institutional case-control cohort of DCIS tissue samples without concurrent or prior IBC, including patients receiving adjuvant therapy (RT and/or ET), that either progressed to ipsilateral or contralateral IBC, or had no evidence of recurrence or progression (DCIS or IBC) for over 10 years. Our aim is to improve our understanding of the molecular events leading to ipsilateral or contralateral IBC occurring at the preinvasive stage of DCIS by performing a comprehensive multi-omic genome-wide survey of their molecular landscapes as well as comparing them with a well-established IBC cohort provided by The Cancer Genome Atlas (TCGA) [19].

While DCIS broadly anticipates the expression, DNA methylation, and copy number patterns that characterize IBC, it exhibits distinctive features as well. All five PAM50 subtypes are represented in our cohort, although the distribution of subtypes in this cohort differs from what is usually seen in IBC. We concur with the observation by Bergholtz and colleagues [20] that characteristic features of Basal IBC are absent in Basal DCIS and provide additional evidence that these diseases may have distinct histories. An analysis of splice-form usage revealed that some DCIS exhibit an unusually low level of splicing complexity and these tumors are associated with increased expression of selected cell-cycle and splice-regulating pathways. Although individual gene level markers demonstrate limited ability to distinguish progressing from non-progressing DCIS, distinct differences at the pathway level offer insight into the development of breast disease.

## Methods

### Sample collection

This study was designed as a multicenter, nested case-control study. Using patient registries at Johns Hopkins Hospital, Baltimore, and University of Alabama at Birmingham in addition to three Surveillance, Epidemiology, and End Results (SEER) Residual Tissue Repository (RTR) centers [21] (University of Southern California, University of Iowa, and University of Hawaii), cases (progressors) and controls (non-progressors) were selected based on initial presentation and required to have a 10-year follow-up. We identified a total of 93 patients with DCIS and no history of prior or synchronous IBC that subsequently progressed to IBC. At the same time, we selected a control group of 93 patients, matched based on race, histological grade, margin status, adjuvant treatments, age (± 5 years) and year of diagnosis (± 5 years), and institution, but who remained recurrence or progression-free for at least 10 years (Supplemental Figure S1, Supplemental Table ST1). In addition, we obtained a total of 11 normal breast tissue samples (7 DCIS-adjacent normal breast, 4 normal breast). All tissues were obtained with each institution’s Institutional Review Board approval. We obtained 20 unstained slides with matching H&E-stained slides from archival formalin-fixed paraffin-embedded (FFPE) tissue blocks. The H&E-stained slides were reviewed and annotated by four study pathologists (Drs. Edward Gabrielson and Ashley Cimino-Mathews, Johns Hopkins; Dr. Paul van Diest, University Medical Center Utrecht; and Dr. Debra Hawes, University of Southern California).

### DNA and RNA isolation

Pathologist-annotated H&E slides were used to guide macrodissection of unstained slides to enrich for > 70% DCIS epithelial cells. Nucleic acids were extracted using AllPrep DNA/RNA FFPE kit (Qiagen, Valencia, CA, USA). Extracted DNA and RNA were quantified by Qubit 2.0 fluorometer (Life Technologies, Carlsbad, CA, USA) and stored at -80 °C.

### Expression analysis

50ng of total RNA were used for library construction using TruSeq RNA Exome kits (Illumina, San Diego, CA, USA; catalog number: RS-301-2001). Libraries were sequenced on an Illumina NextSeq500 for paired-end 75 bp reads. Fastq files were generated using bcl2fastq v2.20.0.422. Reads were aligned to the human genome GRCh38 using the splice-aware STAR aligner v.2.4.2a [22]. Gene-level quantification of expression was performed with the package DESeq2 [23], using GENCODE v.27 as reference annotations.

Unsupervised cluster analysis was performed using consensus clustering with non-negative matrix factorization (NMF), after filtering the expression data to include only the most variable genes. We considered clustering with as few as 2 and as many as 9 groups, selecting the 3-cluster solution as offering the best balance between stability and sparseness.

Gene set enrichment analysis (GSEA) was performed on the Hallmark, Curated (C2) and Oncogenic gene set collections from MSigDB [24], using the Wilcoxon-based gene set test implemented in the *limma* package from Bioconductor [25–27]. We performed PAM50 subtyping with ER-status-balancing, as previously published [28].

We used the xCell webtool (https://comphealth.ucsf.edu/app/xcell) to deconvolve our expression profiles and estimate the relative abundances of 64 cell types in each of our DCIS samples [29].

### Methylation analysis

DNA samples were bisulfite-treated using the EZ DNA Methylation kit (Zymo Research, Irvine, CA, USA). Bisulfite-treated genomic DNA was restored and arrayed using the Illumina Infinium HumanMethylation450K BeadChip Kit (WG-314-1003) in the SKCCC Microarray Core (Johns Hopkins Oncology Center, Baltimore, MD, USA).

GenomeStudio software (Illumina Inc., San Diego, CA, USA) was used to estimate quality control metrics. Quality control metrics were validated through control probe signal intensities extracted using minfi software in R [30]. GenomeStudio-derived detection p-values (threshold of *p* < 0.01) were used to calculate sample-wise call rates. Samples with call rates of < 80% were removed from the analysis. Raw beta values were plotted and samples with atypical beta value plots were removed from the analysis. Probe-wise detection p-values were estimated and probes with > 95% coverage across remaining samples were retained for analysis. Probes with interrogated CpGs 2 bp from a known single nucleotide polymorphism (SNP) with a minor allele frequency (MAF) > 5% were removed.

Data were normalized using the minfi package from Bioconductor (www.bioconductor.org), using the functional normalization algorithm to correct differences between samples [30, 31]. Unsupervised clustering analysis was performed using the ConsensusClusterPlus package [32] from Bioconductor, together with Prediction analysis for microarrays algorithm [33] as implemented in the R package pamr (https://cran.r-project.org/web/packages/pamr/index.html), after filtering the methylation data to include only the most variable probes comparing progressors and non-progressors. We considered clusters with as few as 2 and as many as 9 groups, selecting the 6-cluster solution as offering the best balance between stability and sparseness. Because two of the groups, Methylation Cluster 5 and 6, were very sparse, we combined them for display (Methylation Cluster 5/6). Heatmaps were generated by the R function heatmap and R package (RColorBrewer).

We used MethylResolver to deconvolve our DNA methylation profiles and estimate the relative abundances of 12 cell types in each of our DCIS samples [34].

### DNA Copy Number Analysis

DNA Copy number was estimated from Illumina Infinium HumanMethylation450K data using the Conumee package, R package version 1.9.0 from Bioconductor (https://bioconductor.org/packages/release/bioc/html/conumee.html), using our 11 morphologically normal breast tissue samples as reference [26, 35]. Copy number based subclassification was performed using NMF, calculated with the R package NMF, using the top 10% of genes with the most variable copy number. The R package (CancerSubtypes) was used to facilitate classification [25, 36].

### RNA splicing analysis

We used the MntJULiP program [37] to determine differential splicing events at the intron level and to estimate the splicing ratios of individual introns. Herein an “intron” refers to a segment of the genome between two exons that is excluded from mRNA, as identified from the RNA-seq spliced read alignments. MntJULiP groups all introns corresponding to different and mutually exclusive splice forms that share either their 5’ or the 3’ endpoint. The splicing ratio of each intron in the group, or Percent Splice In (PSI), is defined as the relative contribution of the reads spanning the ith intron to the group’s abundance, expressed as a fraction in the [0,1] interval: PSI (y_i_) = y_i_ / (y_1_ + y_2_ + y_3_), where y_i_ is the number of reads supporting intron i (Supplemental Figure S2). MntJULiP calculates each intron’s abundance y_i_ in each sample from spliced read alignments. It then uses a Dirichlet multinomial distribution coupled with a log likelihood ratio test to identify differences in the group’s splicing ratios between conditions and reports the relative abundance of each variant as the proportion of the variant within the group [37]. Starting from the relative abundance of variants in each splice form group, we further calculated a composite measure of splicing complexity (s) for each group in each sample, based on the difference between the relative abundance of the highest abundance (‘primary’) splice form and the group average, *s = 1- (PSI*_*max*_*- PSI*_*avg*_). A larger *s* value indicates a smaller contribution of the primary isoform to the group, and therefore higher splice form complexity (s).

### Data availability

The DNA methylation and mRNA expression datasets (read counts) generated in this study were deposited in the NCBI Gene Expression Omnibus (GEO) under the GEO accession ID: GSE281303 [transcriptome] and GSE281307 [methylome].

## Results

### Patient characteristics

As seen in Supplemental Table ST1, the 93 progressors and 93 non-progressors were well-balanced with respect to age, year of diagnosis, and race. We obtained complete data sets (i.e., all modalities passed all Q/C) for mRNA expression, DNA methylation, DNA copy number variation, and alternative splicing in 122 of the 186 DCIS samples (see Study Design, Supplemental Figure S1). The median age of non-progressor (control) and progressor (cases) cohorts was 63 and 60 years old, respectively (Supplemental Table ST1). The predominant race in both cohorts was White, while Asian and Hispanic races were rare in both cohorts. Non-progressors, chosen to have at least 10 years of disease-free follow-up, had a median follow-up of 163 months. Progressors advanced to IBC on average 63.6 months (median 59 months) after DCIS diagnosis and progression occurred both ipsilaterally and contralaterally (*n* = 56 and 36, respectively). Nuclear grade was not significantly different between progressors and non-progressors. Progressors and non-progressors had similar rates of RT (48% and 39%, respectively), and ET (16%, in both groups). We were unable to assess differences in lesion size because this information was not available for our cohort.

### Gene expression

#### General characteristics

**Fig. 1:**
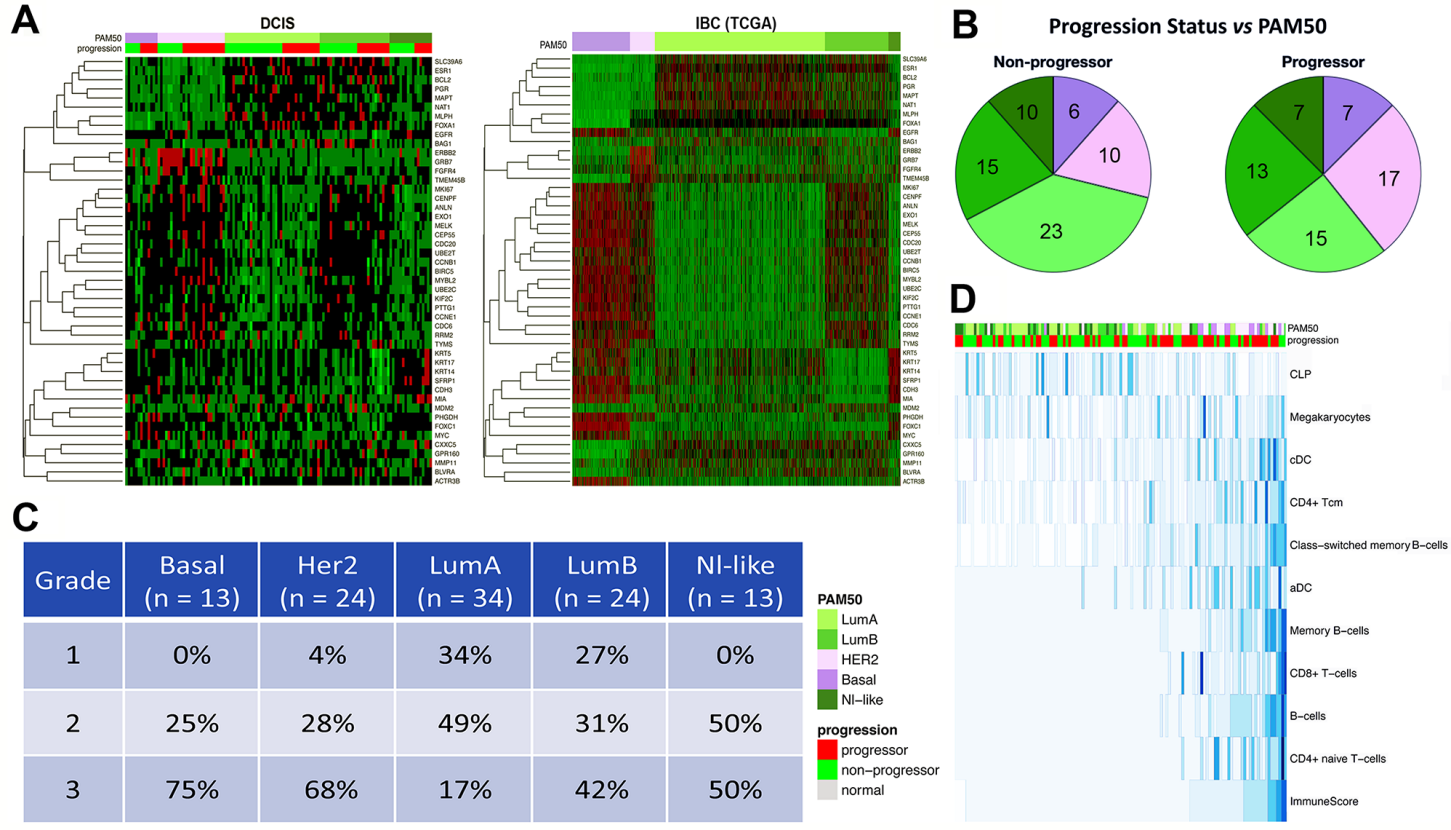
DCIS Gene Expression data results. **A**. Heatmaps showing PAM50 genes and their expression in DCIS samples (left panel) and TCGA-Breast IBC samples (right panel), organized by PAM50 intrinsic subtype (top bar, left-to-right: Basal [purple], HER2 [pink], Luminal A [light green], Luminal B [green], Normal-like [dark green]). The same PAM50 subtype color scheme is used in panels **1A** through **1D**. The green to red gradient indicates increasing expression levels. The lower bar in the left panel shows the DCIS progression status: progressors (red), non-progressors (green). **B.** Pie charts of PAM50 distribution among DCIS samples: Non-progressing DCIS (left pie chart), Progressing DCIS (right pie chart). Sample sizes are shown in each wedge. **C.** Association of DCIS PAM50 intrinsic subtypes with nuclear grade of DCIS. **D.** xCell deconvolution of the DCIS expression data organized by increasing composite ImmuneScore (bottom row). Rows are Z-transformed. Top bar (PAM50) shows intrinsic subtype (shown in the column annotation). Right side lists the identified immune cell types: CLP: common lymphoid progenitor; Megakaryocytes; cDC: conventional dendritic cells; CD4 + Tcm; Class-switched memory B-cells; aDC: plasmacytoid dendritic cells; Memory B-cells; CD8 + T-cells; B-cells; CD4 + naïve T-cells; composite ImmuneScore. Blue gradient indicates relative abundance of each cell type, where higher intensity (darker blue) denotes higher abundance of a particular cell type.

Patterns of expression of PAM50 genes in our DCIS samples broadly match those seen in the TCGA IBC samples (Fig. 1A) [19]. The distribution of subtypes in this cohort of DCIS samples differs from what is usually seen in IBC, with human epidermal growth factor receptor 2 (HER2) tumors relatively over-represented and Luminal A tumors markedly underrepresented in DCIS (Fig. 1A and B).

Expression of estrogen receptor (ER), progesterone receptor (PR), HER2, and Ki67 varies by PAM50 subtype, as expected, with luminal tumors showing high levels of ER and PR expression (Supplemental Figure S3A and S3B), and low levels of HER2 expression compared to HER2-enriched tumors which have the opposite pattern (Supplemental Figure S3C). Furthermore, Basal, HER2, and Luminal B tumors show higher levels of the proliferation marker Ki67 (Supplemental Figure S3D). Likewise, nuclear grade was significantly associated with PAM50 intrinsic subtype, with grade 3 tumors significantly less common in Luminal A DCIS samples than in Basal, HER2, or Luminal B DCIS samples (Fig. 1C). A chi-square test of association between grade and PAM50 was statistically significant (X^2^ = 32.573, df = 8, p-value = 7.351e-05.

Next, we used xCell to deconvolve cellular expression profiles to estimate relative levels of 64 immune and stromal cell types (Fig. 1D), correlating the results with intrinsic subtype [29]. Although we did not observe statistically significant associations, a composite ImmuneScore, summarizing abundance of immune cells, is overall slightly lower in progressors and strongly associates with ER^−^ status. Luminal A progressors had slightly higher levels of immune cells than non-progressors, although the difference was not statistically significant (*p* = 0.135). Luminal B and Basal DCIS had a higher ImmuneScore in non-progressors compared to progressors. HER2-enriched progressors and non-progressors had similar ImmuneScore values. Several immune cell types, including CD4^+^ and CD8^+^ T-cells and memory B cells, were found in greater abundance in ER^−^ DCIS samples. Common lymphoid progenitors (CLP) stood out as a rare cell type with higher levels in ER^+^ tumors (Fig. 1D).

We then performed unsupervised expression cluster analysis on the DCIS samples, which resulted in groups that were enriched in specific PAM50 subtypes (Supplemental Figures S4A, S5A), with Expression Cluster 1 highly enriched for HER2 and Basal tumors, while Expression Cluster 2 consisted mostly of luminal tumors. Expression Cluster 3 consisted of a mixture of all five PAM50 subtypes. Furthermore, unsupervised clustering of the most variable genes in DCIS (Supplemental Figure S4B) also shows resulting clusters to correlate with PAM50 subtypes, a pattern that is even more pronounced when applying the DCIS-derived gene set to an unsupervised clustering of IBC (TCGA) expression data (Supplemental Figure S4C).

### Progressors vs. non-progressors

The HER2 DCIS subtype was more frequent in progressors than in non-progressors (29% vs. 17%, respectively), while Luminal A DCIS were less common in progressors (25% vs. 37%, respectively) (Supplemental Figure S5B), although differences did not reach statistical significance. Gene-level differential expression analysis comparing progressors to non-progressors did not yield statistically significant results (false discovery rate, FDR > 0.10) and a heatmap of the top 100 most differentially expressed genes does not clearly delineate samples by outcome (Supplemental Figure S6). We also explored the differential expression of the 5 proliferation genes included in the ODX-DCIS panel and did not find significant differences between the outcome groups (Supplemental Figure S7) in our RNA-Sequencing-based assessment.

GSEA did highlight several significant differences between DCIS progressors and non-progressors at the pathway level. Several of the Hallmark and Curated (C2) gene sets from the MsigDB collection show differential expression between DCIS progressors and non-progressors in immune pathways as well as in pathways associated with cell cycle and proliferation. Notably, the Hallmark Epithelial-Mesenchymal Transition (EMT) pathway (FDR 0.013) was among the most *up*-regulated pathways in DCIS progressors (Table 1, see Supplemental Table ST2 for a comprehensive list of differentially expressed genes and pathways). Several IBC-associated gene sets also showed significant differential expression, including the POOLA_INVASIVE_BREAST_CANCER_UP gene set (FDR < 0.00001), and the SCHUETZ_BREAST_CANCER_ DUCTAL_INVASIVE_UP gene set (FDR = 0.005), which are significantly *up*-regulated in our progressing DCIS samples compared to non-progressing DCIS samples. Pathways *down*-regulated in DCIS progressors compared to DCIS non-progressors include the TURASHVILI_BREAST_DUCTAL_CARCINOMA_ VS_DUCTAL_NORMAL_DN (FDR < 0.00001) and the CHEN_HOXA5_TARGETS_ 9HR_UP (FDR < 0.00001).

**Table 1:** Selected Up- and down-regulated pathways in progressing vs. non-progressing DCIS

Path-ways	Gene sets	FDR	Expression	Genes
Mobility / Development	CHEN_HOXA5_TARGETS_ 9HR_UP	< 0.00001	Down	AKIRIN1, ALG13, CCNL1, CDKN2AIP, CENPC, CHD9, CLK1, CLK4, CNOT4, FAM13B, FBXO38, JMJD1C, LIG4, LINS1, MAFF, NEMF, NRBF2, PPWD1, PRPF38B, RABGGTB, RBBP6, RBM5, RCHY1, RLF, RSRC2, SFPQ, SLTM, SMCHD1, SNX16, SREK1, SRSF10, STRN3, SUPT20H, TAF1D, THUMPD2, TNFRSF10B, UIMC1, VCPKMT, VEGFA, ZNF280D, ZNF451, ZSCAN16, ZZZ3
	POOLA_INVASIVE_BREAST_ CANCER_UP	< 0.00001	Up	AIM2, ARHGAP25, CD19, CD1E, CD2, CD79A, CEP55, COL11A1, COL5A1, CXCR4, FCMR, HLA-DQA1, HLA-DRB6, IGHG1, IGHV1-69, IGHV3-20, IGHV3-21, IGHV3-23, IGKV1D-13, IGKV1OR2-108, IGLV3-10, IGLV3-19, IGLV4-60, LCK, LGALS9, LOXL1, PLAC8, PRMT2, RHOF, RSAD2, S100P, SLC16A3, SP140, TCL1A, TOP2A, TPX2
	SCHUETZ_BREAST_CANCER_ DUCTAL_INVASIVE_UP	0.00496	Up	BGN, COL11A1, COL1A1, COL1A2, SERPINH1, SPARC
	HALLMARK_EPITHELIAL_ MESENCHYMAL_TRANSITION	0.0137	Up	BGN, COL11A1, COL1A1, COL1A2, COL3A1, COLGALT1, LRP1, MMP14, PCOLCE, PLOD1, PLOD3, SDC4, SERPINH1, SPARC
Signaling	TURASHVILI_BREAST_DUCTAL_ CARCINOMA_VS_DUCTAL_ NORMAL_DN	< 0.00001	Down	BHLHE41, CFAP70, DST, ENOSF1, FAM95C, GPM6B, ITGB8, MAFF, MYBPC1, PLEKHS1, SPIN3, SPRED1, TNFRSF10B, ZNF204P

Gene set expression analysis (GSEA) of differentially expressed genes between DCIS progressors and non-progressors at the pathway level of the Hallmark, Curated (C2), and Oncogenic gene set collections from the Molecular Signatures Database (MSigDB). FDR: false discovery rate. See supplemental table ST2 for complete listing.

### DNA methylation analysis

**Fig. 2:**
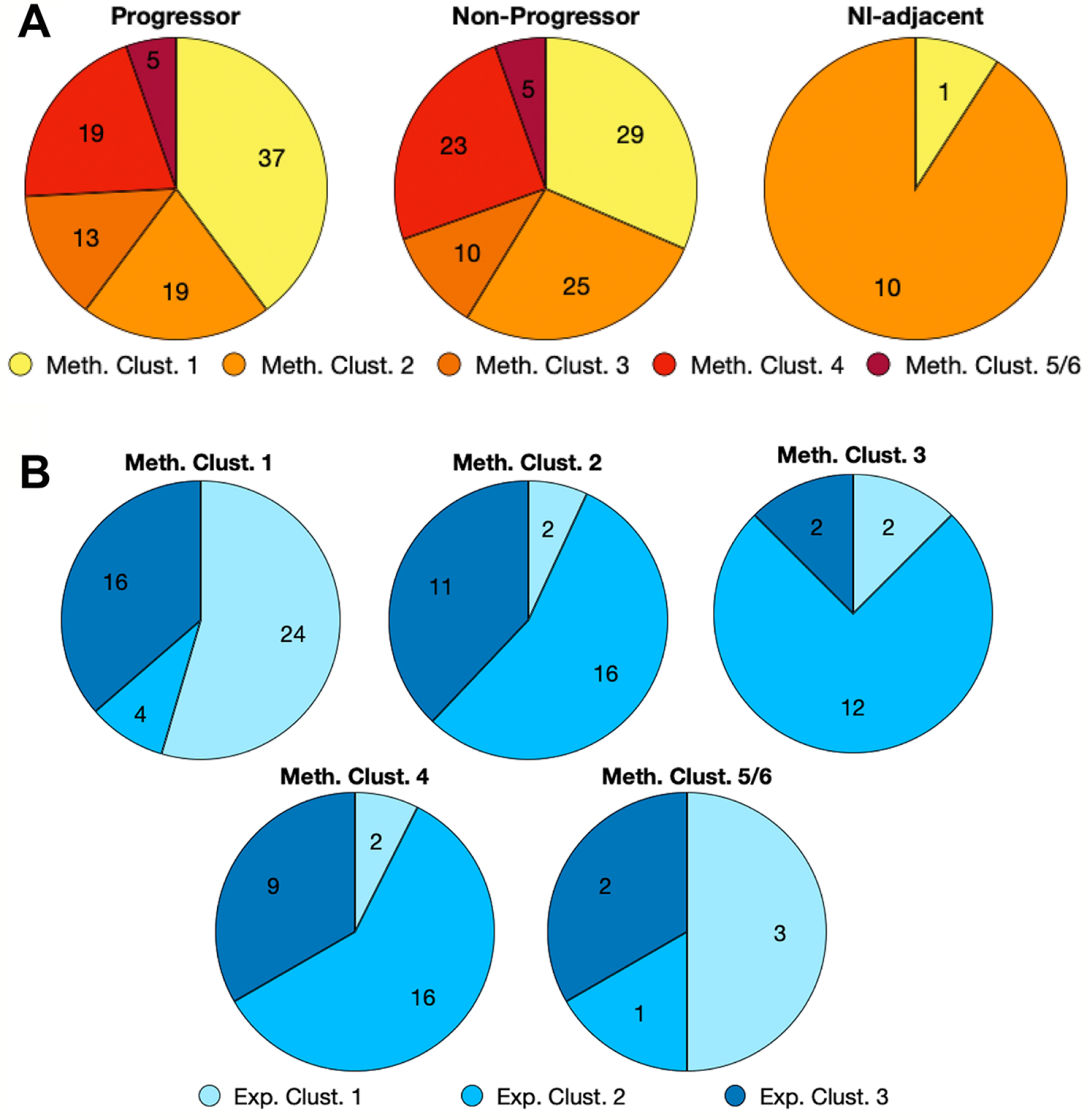
Pie charts showing proportions of DCIS methylation clusters by sample outcome and gene expression clusters. **A.** Methylation cluster proportions of progressors, non-progressors, and normal or DCIS-adjacent normal breast tissue. **B.** Expression cluster proportions of methylation clusters. Sample numbers are shown in each wedge

**Fig. 3:**
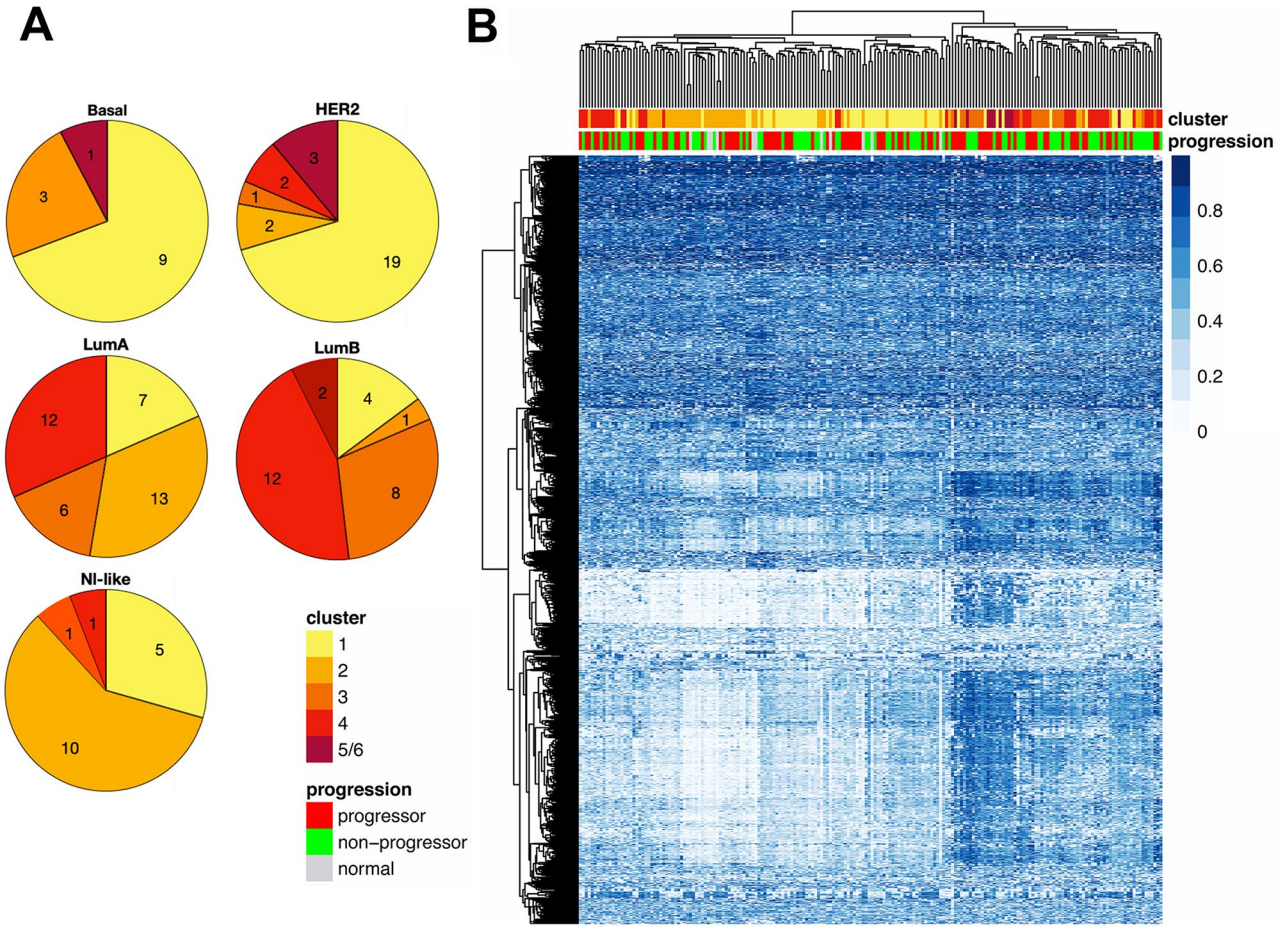
Proportions of DCIS DNA Methylation clusters in PAM50 subtypes. **A.** Pie charts showing methylation cluster proportions of PAM50 intrinsic subtypes: Basal; HER2; LumA: Luminal A; LumB: Luminal B; Nl-like: normal-like. Number of samples is shown in each wedge. **B.** Heatmap showing a hierarchical cluster analysis of the top 0.5% (2370) most variable CpG sites. Top bar shows methylation cluster number, and bottom bar shows progression status. Blue gradient represents proportion of methylated CpG (beta value) where higher intensity (darker blue) denotes higher methylation

**Fig. 4:**
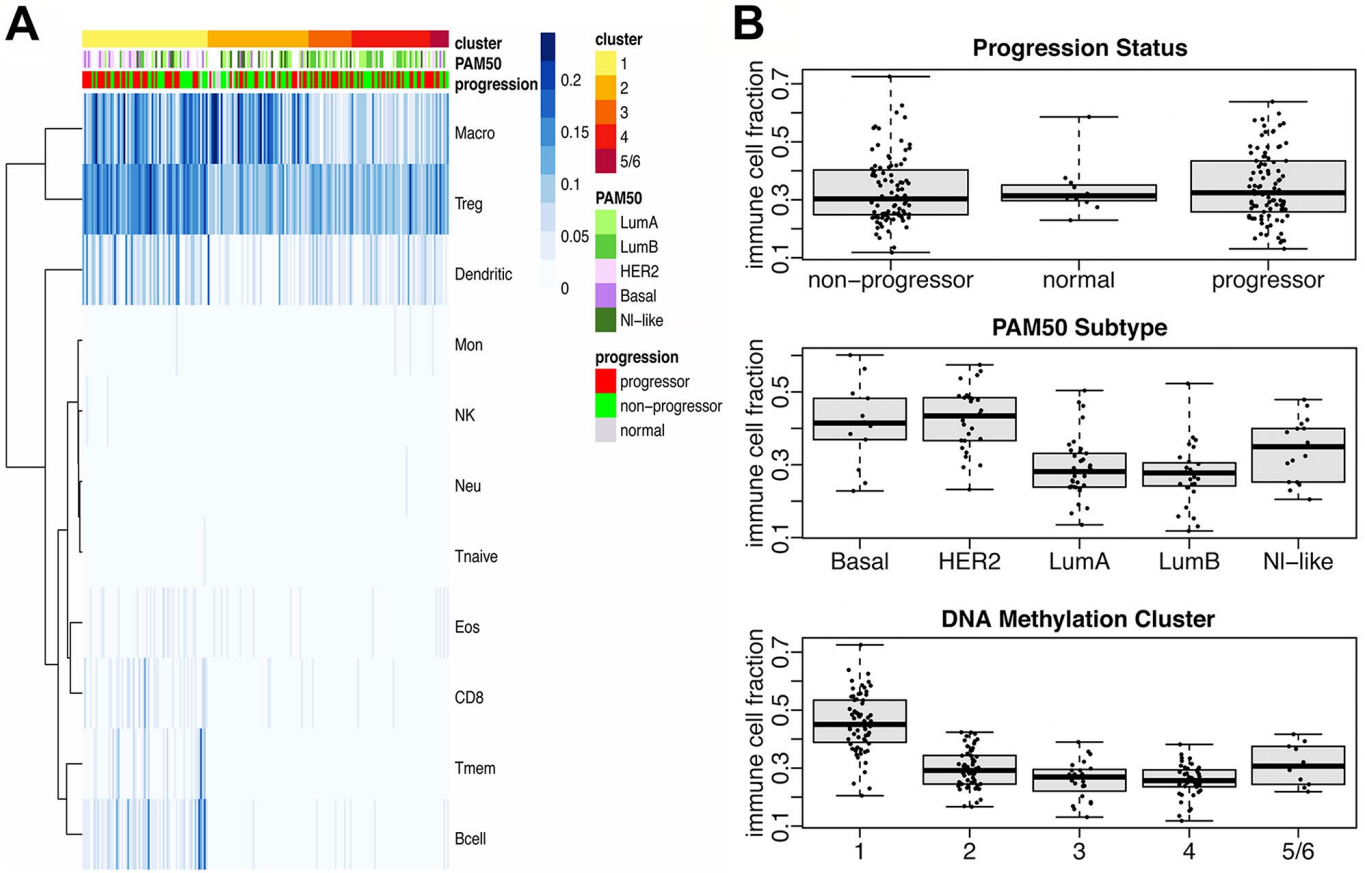
Analysis of immune cell infiltration by methylation cluster, PAM50, and progression status. **A.** Heatmap of immune cell infiltration organized by methylation cluster (top bar). PAM50 (middle bar, samples missing PAM50 data are indicated in white), and progression status (bottom bar) are shown as annotations. Immune cell infiltration is shown as proportion of cells present (blue gradient), where darker blue denotes greater proportion of cells. Identified immune cell types: Macro: Macrophages; Treg: Regulatory T cells; Dendritic: Dendritic cells; Mon: Monocytes; NK: Natural killer cells; Neu: Neutrophils; Tnaive: Naïve T cells; Eos: Eosinophils; CD8; Tmem: Memory T cells; B-cells. **B.** Sample immune cell fraction (ICF) and progression status, PAM50 intrinsic subtype, and DNA methylation clusters. ICF is a total/aggregate of all immune cells shown in the panel A heatmap. Boxplots illustrate the median and the first and third quartiles (box); the whiskers denote range of values. ANOVA: ICF by Progression (F-statistic = 0.33, df = 2, *p* = 0.72); ICF by PAM50 (F-statistic = 15.62, df = 4, *p* = 2.879e-10); ICF by Methylation Cluster (F-statistic = 58.81, df = 4, *p* < 2.2e-16)

Unsupervised cluster analysis was performed using consensus clustering together with the partitioning around medoids algorithm (rPAM), after filtering the methylation data to include only the most variable genes in progressors and non-progressors, resulting in 5 sample groups. Progressors and non-progressors were represented almost equally in all 5 methylation clusters (Fig. 2A). Most of the morphologically normal samples fell into Methylation Cluster 2. The relationships between methylation clusters and expression clusters were explored in Fig. 2B and Supplemental Figure S5D. Expression Cluster 1 dominates Methylation Cluster 1 (Fig. 2B), while Expression Cluster 2 samples represent the predominant fraction in Methylation Clusters 2,3 and 4. The proportions of DCIS methylation clusters in the PAM50 subtypes were shown in Fig. 3. Methylation Cluster 1 was predominant in the Basal and HER2^+^ PAM50 subtypes, while the luminal subtypes were distributed across Methylation Clusters 1 through 4 (Fig. 3A and Supplemental Figure S5C). In Fig. 3B, a hierarchical cluster analysis of the top 0.5% (*n* = 2370) most variable CpG sites did not show DNA methylation in progressors to be consistently different from non-progressors. The methylation clusters did show correlation with infiltration by several immune cell subtypes (Fig. 4A). Samples in Methylation Cluster 1, which was enriched for usually hormone negative cancers (HER2 and Basal PAM50 subtypes), showed high levels of macrophages, T-regulatory cells, dendritic cells, T-memory cell, and B-cells. Methylation Cluster 2, which includes most of the PAM50 normal-like samples as well as true normal (Supplemental Figure S5C, S5E), had high levels of macrophages. We further assessed the total immune cell fraction (proportion of immune cells present in the sample) by outcome, PAM50, and Methylation Cluster (Fig. 4B). The immune cell fraction was similar in progressing DCIS and non-progressing DCIS, but higher in Basal and HER2 DCIS subtypes, which was also reflected in Methylation Cluster 1, which predominated the hormone negative DCIS samples.

**Fig. 5:**
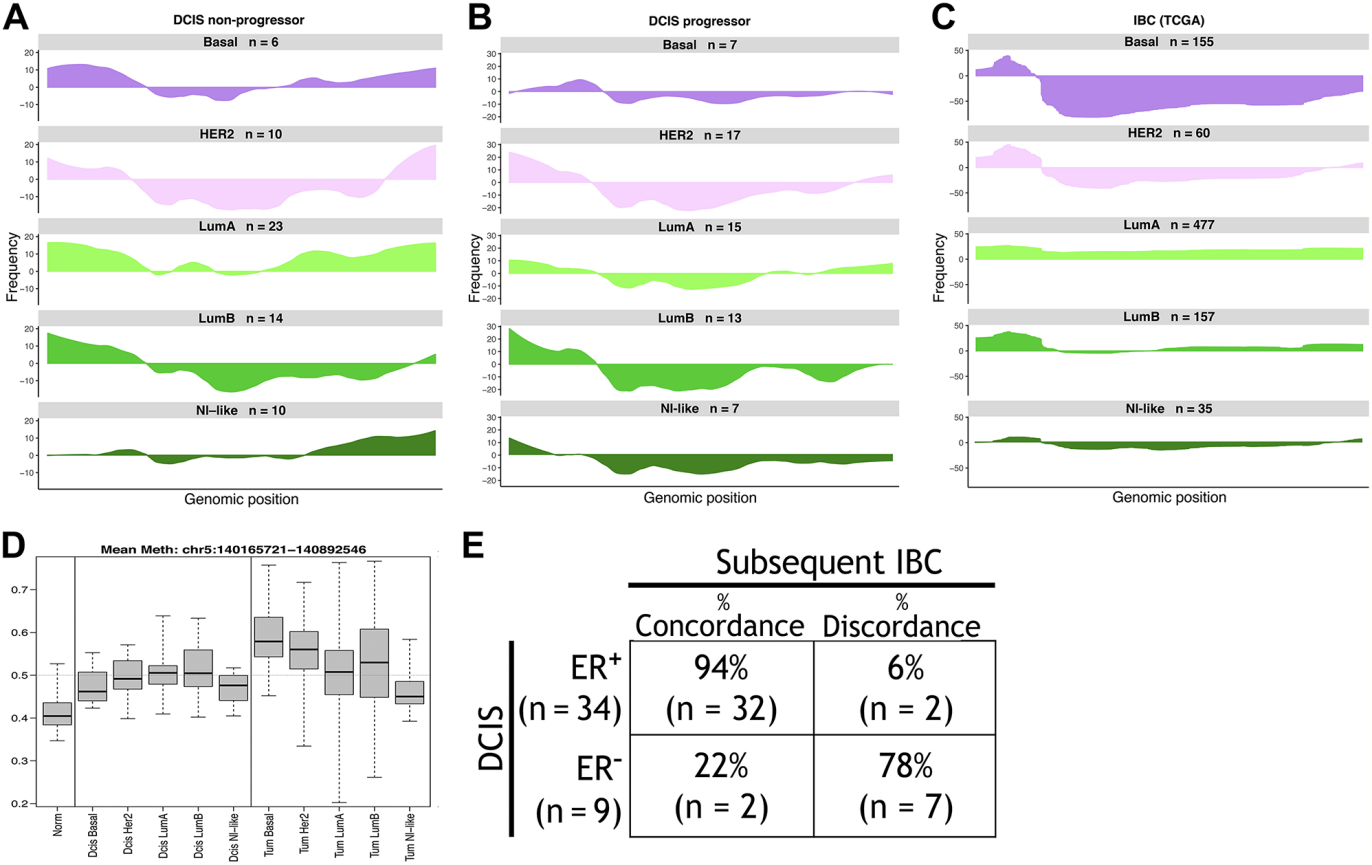
Basal DCIS compared to basal IBC. **A-C.** Frequency-plots of copy number variation (CNV) data at chromosome 5. The PAM50 subtype and number of samples (n) is indicated above each plot. Genomic position is indicated on the x-axis, with p-arm on the left, q-arm on the right. The y-axis shows the frequency of deletions (below baseline) or amplifications (above baseline) in DCIS samples (A. Non-progressors; B. Progressors; C. TCGA-IBC). Note different frequency scales, adapted to the largest %. of deletions or amplifications for each plot. **D.** Methylation of Procadherin genes in DCIS (left) and IBC (right, TCGA), compared to normal tissue (first column), by PAM50 subtype. Boxplots illustrate the median and the first and third quartiles (box); the whiskers denote range of values. **E.** Concordance and discordance ER status of hormone positive or negative DCIS progressing to hormone positive or negative IBC in the same patient. A Fisher’s exact test comparing ER status in DCIS and subsequent cancer was statistically significant (Odds Ratio = 45.41 95%, CI = 5.04-753.63, p-value = 3.636e-05.

Bergholtz and colleagues recently reported in a cross-sectional study of DCIS progression that some molecular hallmarks of Basal subtype IBC are not seen in Basal DCIS [20], raising questions about the natural progression of Basal DCIS. The authors highlight two features of Basal IBC that are absent in Basal DCIS: deletion of the entire q-arm of chromosome 5 and hypermethylation of Procadherin genes on chromosome 5. Our results support both findings (Fig. 5A-C). Specifically, we observed a low rate of deletion in Basal DCIS at chromosome 5q31(~ 2–5%) while this same region had a high rate of deletion among TCGA Basal IBC. We further assessed CNV across all chromosomes for subtype-specific signatures (Supplemental Figure S8). We found another Basal IBC-specific pattern, but of amplification, on Chromosome 2p.

Overall, CNV patterns between DCIS progressors, DCIS non-progressors, and TCGA IBC were often similar, although they tended to be more prominent in IBC than DCIS, e.g., see Chromosome 1 (Supplemental Figure S8). In some cases, e.g., Chromosomes 11 and 13, similar CNV patterns and frequencies were seen in progressors, non-progressors, and IBC across intrinsic subtypes. We were also able to partially confirm the report that Chromosome 3p losses were more frequent in DCIS than in IBC [38], although that appeared to be primarily driven by the more common Luminal A tumors, while in our cohort, Basal and HER2 DCIS showed lower 3p losses than the corresponding TCGA IBC.

Furthermore, for the subset of cases for which we have the necessary information, we calculated concordance and discordance between ER expression in DCIS and in the subsequent IBC. We observed that only 22% of ER^−^ DCIS cases progress to ER^−^ IBC, while 94% of ER^+^ DCIS cases progress to ER^+^ IBC (Fig. 5D).

### Alternative splicing in DCIS

While our data reveal substantial variation in splicing complexity (s) across our DCIS cohort, the variation was not significantly associated with risk of progression, PAM50 intrinsic subtypes, or other clinical or pathologic variables (Supplemental Figure S9A-E). The expression cluster result, on the other hand, was highly statistically significant (*p* < 0.00001, Supplemental Figure S9C). Expression Clusters 1 and 2, associated with ER^−^ and ER^+^ disease, respectively, exhibit low complexity, while Expression Cluster 3, which includes a mix of PAM50 subtypes, contained most of the high-complexity tumors. Although s-scores show some variation across PAM50 intrinsic subtypes, with Basal tumors exhibiting the highest median complexity, and Luminal A the lowest, the differences did not reach statistical significance by ANOVA (Supplemental Figure S9B). Moreover, some variation was evident across methylation clusters, but it did not reach statistical significance (Supplemental Figure S9D). Furthermore, splice complexity did not significantly vary with patient age (Supplemental Figure S9E).

Detection of rare splice forms depends on adequate sequencing depth, so we considered the possibility that samples exhibiting low levels of splice complexity across the genome are simply those with the lowest read counts. While it is true that samples with low read counts are more likely to have low s-scores (Supplemental Figure S10A), we also observed that some low complexity samples showed high read counts and some high complexity samples showed low read counts, suggesting that some of the variation we observed in complexity may instead be due to differences in DCIS splice regulation. To identify genes with roles in splice regulation, we considered two sources: (1) the Gene Ontology Database [39], which includes a variety of spliceosome annotations, and (2) the HUGO gene nomenclature group, which maintains a database of genes in the major spliceosome groups, along with mappings onto individual spliceosomal complexes (https://www.genenames.org/data/genegroup/#!/group/1518).

We sought to identify genes that modify splicing in these samples, expecting that splicing complexity will vary with expression of those genes. Accordingly, aggregate s-scores for each sample were correlated, across samples, with levels of gene expression for each gene. Most genes show a positive Spearman correlation between expression and complexity, explained in part by variation in read depth, as low counts limit our ability to see both rare splice variants and low expressing genes. Genes showing a negative correlation coefficient, however, are noteworthy, as they are not readily explained by variation in coverage. A heatmap of s-scores for the intron groups demonstrating the most splice variation across samples illustrates that some samples show consistently lower or higher complexity than others (Supplemental Figure S10C), while the overall expression levels of the same genes show little difference between samples and therefore do not explain why some samples have consistently anti-correlated s-scores (Supplemental Figure S10D).

**Fig. 6:**
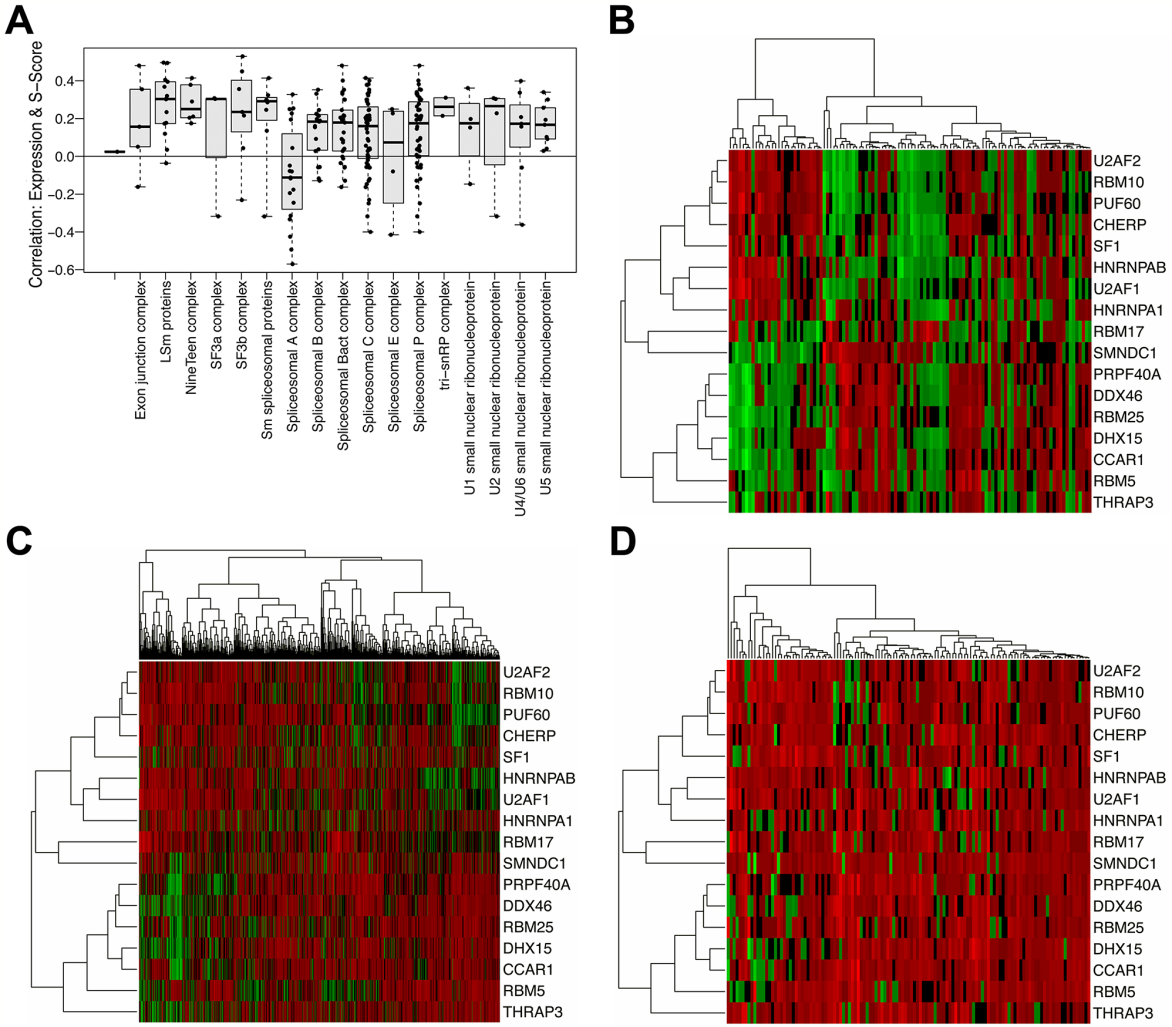
Associations between splice complexity and spliceosomal gene expression. **A.** Spearman correlation between gene expression levels and s-scores (complexity) of HUGO-defined spliceosome complexes in DCIS. Boxplots illustrate the median and the first and third quartiles (box); the whiskers denote range of values. Horizontal line at 0 denotes baseline. ANOVA comparing aggregate sample complexity by spliceosome complex was statistically significant (F-statistic = 32.19, df = 1, p- = 9.73e-08. **B.** Heatmap showing Spliceosome Complex A gene expressions in TCGA normal breast tissue (adjacent to breast cancers). Rows represent genes in Spliceosome Complex A, the green to red gradient indicates increasing expression levels. **C.** Heatmap showing Spliceosome Complex A gene expression in TCGA invasive breast cancer. Rows represent genes in Spliceosome Complex A, in the same order as in heatmap B. **D.** Heatmap showing Spliceosome Complex A gene expression in DCIS. Rows represent genes in Spliceosome Complex A, in the same order as in heatmaps B and C.

A GSEA analysis of anti-correlated gene ontology classes showed that while the most anti-correlated gene ontology classes were associated with cell cycle and cell-replication (Supplemental Table ST3), complexity was also significantly anti-correlated with the expression of genes in several splicing-related ontology classes (Table 2). In most cases, the expression of HUGO spliceosome complex genes was positively correlated with splicing complexity, but the Spliceosomal A complex stood out as it showed an opposite relationship (Fig. 6A). To assess how the expression patterns of spliceosome genes in our DCIS samples compare to normal breast tissue and IBC, we used TCGA IBC-adjacent normal breast samples as well as TCGA IBC data. Specifically, looking at the adjacent normal samples from TCGA, the genes in the Spliceosome A complex were expressed in two clear clusters of genes (Fig. 6B). In both TCGA IBC and our DCIS samples, we observed less organization and the co-expression patterns in the genes are less well defined (Fig. 6C-D).

**Table 2:** Gene ontology RNA splicing-related pathways significantly anti-correlated with complexity (s)

Pathway	FDR	*N*
GOBP_RNA_SPLICING	< 0.00001	470
GOBP_RNA_SPLICING_VIA_TRANSESTERIFICATION_REACTIONS	< 0.00001	377
GOBP_REGULATION_OF_RNA_SPLICING	0.00003	144
GOBP_REGULATION_OF_MRNA_SPLICING_VIA_SPLICEOSOME	0.00014	100
GOBP_ALTERNATIVE_MRNA_SPLICING_VIA_SPLICEOSOME	0.0004	73
GOBP_SPLICEOSOMAL_COMPLEX_ASSEMBLY	0.0024	74
GOCC_SPLICEOSOMAL_COMPLEX	0.00318	187
GOBP_REGULATION_OF_ALTERNATIVE_MRNA_SPLICING_VIA_ SPLICEOSOME	0.00411	57
GOBP_MRNA_SPLICE_SITE_SELECTION	0.02011	46

N: number of genes in each pathway; FDR: false discovery rate. See supplemental table ST3 for complete listing of correlations and anti-correlations between pathways and splice complexity.

## Discussion

DCIS remains a clinical challenge because we still cannot reliably distinguish indolent cases from those likely to progress to IBC. Current methodologies for predicting DCIS recurrence, such as the Oncotype DX^®^ DCIS Score and the DCISionRT^®^ assay, have been derived from low-risk cohorts eligible for surgical treatment only, limiting the discovery of high-risk molecular signatures, since withholding adjuvant treatment in high-risk cases cannot be justified. In this study, our choice to include such cases came with the limitation that outcomes were very likely influenced by individual treatment choices, including adjuvant RT and ET, which decreased the likelihood of discovering outcome-predictive molecular signatures. Therefore, our focus was to begin the process of mapping out the molecular and genetic landscapes of DCIS, with the purpose of identifying molecular subtypes and their key drivers in this disease.

The landmark TCGA breast study [40] documented that breast cancer is extremely heterogenous. The two dominant mutations (PIK3CA and TP53) only account for ~ 40% of cases. This is in contrast to cancers such as colon cancer whose dominant mutations can account for 60 − 80% of cases [41]. The overarching goal of our large-scale DCIS survey was to utilize genome wide analyses to help categorize the heterogenous biology of DCIS.

Comparing our DCIS cohort to TCGA IBC at the gene expression level, we broadly observed similar patterns (Fig. 1A, Supplemental Figures S4B, S4C). Our DCIS outcome analyses comparing gene expression (Fig. 1A, Supplemental Figure S6) and DNA methylation (Fig. 3B) did not show significant differences between DCIS progressors and non-progressors, nor did molecular subclasses based on PAM50 subtype (Fig. 1A and B, Supplemental Figure S5B), gene expression clusters (Supplemental Figure S5F) or methylation clusters (Supplemental Figure S5E). While we did observe subtle differences in molecular subsets, such as that hormone negative DCIS (Basal and most HER2 subtypes) are more likely to be progressors compared to hormone positive DCIS (Supplemental Figures S5B, S5E, S5F), these results would be insufficient to predict progression to IBC, although this may be due in part to the increasingly small sample numbers in the molecular subclasses.

Interestingly, we did observe significant differences between DCIS progressors and DCIS non-progressors in our GSEA, showing differential expression patterns in pathways that have been associated with disease progression. Notably, the Epithelial-to-Mesenchymal Transition (EMT) pathway (FDR 0.013) was among the most upregulated pathways in DCIS progressors (Table 1, Supplemental Table ST2). This pathway is implicated in cancer progression and metastasis when epithelial cells lose cell polarity and cell-cell adhesion [42]. Furthermore, several IBC-associated gene sets were among the most significantly upregulated in progressors compared to non-progressors. Among these, the POOLA_INVASIVE_BREAST_CANCER_UP and the SCHUETZ_BREAST_CANCER_DUCTAL_INVASIVE_UP gene sets were significantly upregulated in our progressing DCIS compared to non-progressing DCIS. Poola et al. derived these genes by comparing atypical ductal hyperplasia, considered a precursor to DCIS, with and without accompanying invasive disease [43], and Schuetz performed a study that compared DCIS and their matched IDC [44].

Furthermore, several pathways are significantly downregulated in DCIS progressors compared to DCIS non-progressors, including the TURASHVILI_BREAST_DUCTAL_CARCINOMA_VS_DUCTAL_NORMAL_DN gene set (FDR < 0.00001). This gene set was derived in a study that compared gene expression profiles of ductal carcinoma cells and normal ductal cells, which showed this gene set to be downregulated in cancer cells [45]. Another gene set, CHEN_HOXA5_TARGETS_9HR_UP, is also significantly downregulated in our DCIS progressor (FDR < 0.00001). This gene set was derived from a study utilizing microarray analysis of an inducible HOXA5 breast cancer cell line (HS578T) to identify genes whose expressions are modified after HOXA5 induction [46]. HOXA5 is a homeotic gene and tumor suppressor known to play a role in tumorigenesis [47]. GSEA further highlighted differences in immune pathways and pathways associated with cell cycle and proliferation (Supplemental Table ST2).

Bergholtz and colleagues recently reported that several characteristics of Basal invasive ductal breast tumors are not seen in Basal DCIS, questioning Basal DCIS as precursor lesions to Basal invasive breast carcinoma [20]. We observed similar findings in our DCIS cohort and provide additional support to this view. Our CNV analysis confirms a lack of widespread deletion of Chromosome 5q in Basal DCIS, while this pattern is commonly observed in Basal TCGA breast cancers (Fig. 5A-B). It is possible that this deletion is a necessary event for DCIS to progress to Basal IBC. Our data also demonstrated lack of hypermethylation of Procadherin genes in Basal DCIS, a pattern that is seen in the Basal IBC samples from TCGA (Fig. 5C).

In addition, an analysis of ER concordance in our progressor cohort showed that hormone positive DCIS most often progressed to hormone positive IBC (94%), whereas hormone negative DCIS usually progressed to hormone positive IBC (78%, Fig. 5D).

Our comprehensive analysis of copy number variation (CNV) patterns in DCIS non-progressors, progressors, and IBC across each PAM50 subtype (Supplemental Figure S8) revealed several patterns of interest, although these must be interpreted with caution considering the small sample numbers available for some subsets (see Fig. 5A). Overall, CNV patterns in DCIS and IBC were often similar, in many instances even PAM50 subtype-specific, including a trend that the frequency of a given CNV increased from non-progressing DCIS to progressing DCIS to IBC, e.g., the amplification on Chr. 1q. Other patterns are more difficult to reconcile with progression, e.g., deletions in Chr. 21p seen in 20% of our Luminal A and HER2 DCIS progressors, but largely absent in the respective IBC.

Finally, we characterized differences in alternate gene splicing because disruption of normal regulatory processes during tumor development and progression is well documented to drive widespread changes in splice form usage [48–50], and may result in a genome-wide increase or decrease in splicing complexity in individual tumors. Although our data revealed substantial variation in splice complexity across DCIS samples, that variation was not significantly associated with the clinicopathologic variables studied.

One limitation to this study is the lack of immunohistochemically determined hormone status on many samples, which would have provided a more definitive answer whether hormone negative DCIS are precursors to hormone negative IBC. DCIS patient-matched IBC were not available in this study; we did, however, have the clinical reports and long-term follow-up for all study samples.

## Conclusions

In summary, while our study has demonstrated numerous molecular differences between DCIS progressors and DCIS non-progressors, their interpretation and potential usefulness as predictive outcome tools are complicated by the heterogeneity of the disease, and its balkanization into multiple subtypes, each with their own molecular alterations, immunological profiles, and susceptibility to treatment options. While the resulting low sample numbers in individual subsets currently prevents robust conclusions, several findings and associations suggest promising avenues for further studies. Of particular interest are the highly significant associations with IBC progression of several gene sets revealed by our GSEA. Some of these may lend themselves to the development of a prognostic molecular score, to be validated on independent DCIS cohorts.

## Electronic supplementary material

Below is the link to the electronic supplementary material.

Supplementary Material 1

Supplementary Material 2

Supplementary Material 3

Supplementary Material 4

Supplementary Material 5

Supplementary Material 6

Supplementary Material 7

Supplementary Material 8

Supplementary Material 9

Supplementary Material 10

Supplementary Material 11

Supplementary Material 12

Supplementary Material 13

Supplementary Material 14

Supplementary Material 15

## Data Availability

The DNA methylation and mRNA expression datasets (read counts) generated in this study were deposited in the NCBI Gene Expression Omnibus (GEO) under the GEO accession ID: GSE281303 [transcriptome] and GSE281307 [methylome].Additionally, Data is provided within the supplementary information files.

## Abbreviations

DCIS: Ductal carcinoma in situ
IBC: Invasive breast cancer
RT: Radiation therapy
ET: Endocrine therapy
ER: Estrogen receptor
IBR: In breast recurrence
TCGA: The Cancer Genome Atlas
SEER RTR: Surveillance, Epidemiology, and End Results Residual Tissue Repository
FFPE: Formalin-fixed paraffin-embedded
NMF: Non-negative matrix factorization
GSEA: Gene set enrichment analysis
SNP: Single nucleotide polymorphism
MAF: Minor allele frequency
PSI: Percent splice-in
s: Splicing complexity
GEO: Gene Expression Omnibus
HER2: Human epidermal growth factor receptor 2
PR: Progesterone receptor
CLP: Common lymphoid progenitors
FDR: False discovery rate
EMT: Epithelial-mesenchymal transition
CNV: Copy number variation
Chr: Chromosome
