# Supplementary material for: Multimodal genome-wide survey of progressing and non-progressing breast ductal carcinoma in-situ
Source: Breast Cancer Res. 2024 Dec 4;26:178. doi: 10.1186/s13058-024-01927-1 (PMC11616160; doi:10.1186/s13058-024-01927-1)
Supplement: Supplementary file 1 — Supplementary Material 1 [file 13058_2024_1927_MOESM1_ESM.docx]

**Supplementary Table 1.** Study population characteristics.

**Supplementary Table 2.** GSEA of DCIS progressors and non-progressors.

Gene set expression analysis (GSEA) of differentially expressed genes between DCIS progressors and non-progressors at the pathway level of the Hallmark, Curated (C2), and Oncogenic gene sets. The most differentially expressed genes (top 5%) that overlap each pathway are shown in the Genes column. N: number of genes in pathway. AvgExpr: Average log of gene expression. logFC: Log of fold-change of gene expression between DCIS progressors and non-progressors. FDR: False discovery rate.

**Supplementary Table 3.** Correlation of Gene Ontology Pathways and splice complexity.

Gene ontology pathway analysis showing the most correlated and anti-correlated pathways between splice complexity (s) and gene expression. N: number of genes in pathway. FDR: False discovery rate.
