# Supplementary material for: Multimodal genome-wide survey of progressing and non-progressing breast ductal carcinoma in-situ
Source: Breast Cancer Res. 2024 Dec 4;26:178. doi: 10.1186/s13058-024-01927-1 (PMC11616160; doi:10.1186/s13058-024-01927-1)
Supplement: Supplementary file 2 — Supplementary Material 2 [file 13058_2024_1927_MOESM2_ESM.docx]

**Supplementary Table 1.** Selected characteristics of the study population.

|  | Non-progressing DCIS (n=93) | Progressing DCIS (n=93) |
| --- | --- | --- |
| Age range (yrs.) | 34 – 87 | 29 – 92 |
| Median | 63 | 60 |
| Year Diagnosis (median) | 1998 | 1999 |
| Race |  |  |
| Asian/Other | 2 | 2 |
| Black | 6 | 6 |
| Hispanic | 2 | 3 |
| White | 83 | 82 |
| Follow-up / Time-to-Event (mo.) | 120 – 319 | 9 – 192 |
| Median | 163 | 59 |
| Sidedness |  |  |
| Contralateral | ----- | 36 |
| Ipsilateral | ----- | 56 |
| Unknown | ----- | 1 |
| Nuclear Grade |  |  |
| Grade 1 | 13 | 19 |
| Grade 2 | 33 | 31 |
| Grade 3 | 44 | 40 |
| Unknown | 3 | 3 |
| Radiation Therapy |  |  |
| Yes | 34 | 41 |
| No | 53 | 45 |
| Unknown | 6 | 7 |
| Hormone Therapy |  |  |
| Yes | 14 | 14 |
| No | 73 | 72 |
| Unknown | 6 | 7 |
