# Supplementary material for: Multimodal genome-wide survey of progressing and non-progressing breast ductal carcinoma in-situ
Source: Breast Cancer Res. 2024 Dec 4;26:178. doi: 10.1186/s13058-024-01927-1 (PMC11616160; doi:10.1186/s13058-024-01927-1)
Supplement: Supplementary file 5 — Supplementary Material 5 [file 13058_2024_1927_MOESM5_ESM.docx]

**Supplemental Figure Legends for: Multimodal Genome-wide Survey of Progressing and Non-progressing Ductal Carcinoma In-Situ**

**Figure S1.** **Study design**. Using patient registries at Johns Hopkins Hospital, UAB, and SEER RTR, we identified 93 DCIS patients progressing to invasive breast cancer (IBC) and 93 DCIS patients with no recurrent disease within a follow-up period of at least 10 years. The 2 cohorts were matched by age, race, and year of diagnosis. Numbers indicate available samples yielding sufficient RNA & DNA and passing quality controls of the assays used. JHH: Johns Hopkins Hospital; UAB: University of Alabama at Birmingham; U. Iowa: University of Iowa; USC: University of Southern California; U. Hawaii: University of Hawaii; P: progressors; NP: non-progressors; N: normal or adjacent normal breast tissue samples.

**Figure S2.** **Splice complexity equations**. Percent Splice In (PSI) is defined as the relative contribution of the reads spanning the i^th^ intron to the group’s abundance. The splice complexity (s) of a group equals the difference between the relative abundance of the highest abundance splice form and the group average:

*s = 1- (PSI_max_ - PSI_avg_*).

**Figure S3.** **Validation of PAM50 intrinsic subtyping in DCIS**. **A-D** Box plots of ER, PR, HER2, and Ki67 expression levels, respectively. Boxplots illustrate the median and the first and third quartiles (box); the whiskers denote range of values. LumA: Luminal A; LumB: Luminal B; Nl-like: Normal-like.

ANOVA: ER by PAM50 (F-statistic = 47.18, df = 4, p = < 2.2e-16); PR by PAM50 (F-statistic = 26.42, df = 4, p = 1.187e-15); HER2 by PAM50 (F-statistic = 21.97, df = 4, p = 1.426e-13); KI67 by PAM50 (F-statistic = 16.43, df = 4, p = 9.955e-11).

**Figure S4.** **Relation of PAM50 Intrinsic subtypes and Expression Clusters**. **A.** Pie charts showing intrinsic subtype composition of each expression cluster. Sample sizes are shown in each wedge. A chi-square test of association between Expression Cluster and PAM50 was statistically significant, X^2^ = 70.767, df = 8, p-value = 3.457e-12. **B.** Unsupervised clustering of DCIS expression data of the most variable genes (sd > 2.5, n= 2045). 1237 of these were also measured in the TCGA data and are shown here. **C.** Unsupervised clustering of TCGA (IBC) expression data using the DCIS-derived gene set. The row order (genes) is from the DCIS plot and reproduced in the TCGA data. Bars above the heat maps show PAM50 intrinsic subtypes as indicated. The lower bar on the DCIS heatmap shows progressors (red) vs non-progressors (green).

**Figure S5. Sample distributions in different classification modalities.**

**A.** PAM50 vs Expression Clusters. Expression Clusters 1-3 are shown as columns and PAM50 intrinsic subtypes as rows. Each cell shows the number of samples (N), the %-fraction of the PAM50 intrinsic subtype, and the %-fraction of expression cluster this represents. The pie charts in the bottom row show the PAM50 subtype fractions in each Expression Cluster. The pie charts in the last column show the Expression Cluster fractions in each PAM50 subtype.

**B.** PAM50 vs Outcomes. Outcomes are shown as columns and PAM50 intrinsic subtypes as rows. Each cell shows the number of samples (N), the %-fraction of the PAM50 intrinsic subtype, and the %-fraction of samples in each outcomes group. The pie charts in the bottom row show the PAM50 intrinsic subtype fractions for each Outcome. The pie charts in the last column show the outcome fractions in each PAM50 intrinsic subtype. NP: non-progressor; P: progressor.

**C.** PAM50 vs Methylation Clusters. Methylation Clusters 1-6 are shown as columns and PAM50 intrinsic subtypes as rows. Each cell shows the number of samples (N), the %-fraction of PAM50 intrinsic subtype, and the %-fraction of samples in each methylation cluster. The pie charts in the last row of each column show the PAM50 intrinsic subtype fractions in each Methylation Cluster. The pie charts in the last column show Methylation Cluster fractions in each PAM50 subtype.

**D.** Methylation Clusters vs Expression Clusters. Methylation Clusters 1-6 are shown as columns and Expression Clusters as rows. Each cell shows the number of samples (N), the %-fraction of expression clusters this represents, and the %-fraction in each methylation cluster. The pie charts in the last row show Expression Cluster fractions in each Methylation Cluster. The pie charts in the last column show Methylation Cluster fractions in each expression cluster.

**E.** Methylation Clusters vs Outcomes. Methylation Clusters 1-6 are shown as columns and Outcomes (Normal: normal & adjacent normal breast) as rows. Each cell shows the number of samples (N), the %-fraction in each outcomes group, and the %-fraction in each Methylation Cluster. Pie charts in the last row show Outcomes in each Methylation Cluster. The pie charts in the last column show Methylation Cluster fractions in each Outcome.

**F.** Expression Clusters vs Outcomes. Outcomes are shown as columns and Expression Clusters as rows. Each cell shows the number of samples (N), the %-fraction in each Expression Cluster, and the %-fraction for each Outcome. Pie charts in the last row show Expression Cluster fractions for each Outcome. The last column shows the Outcomes for each Expression Cluster.

**Figure S6.** **DCIS gene expression data**. Heatmap showing gene expression cluster analysis of top 100 most differentially expressed genes between DCIS progressors (red) and non-progressors (green) (top bar). Rows represent genes in this analysis.

**Figure S7: ODX-DCIS proliferative gene expression in DCIS.** Quantitation of gene-level expression of the 5-gene proliferation group of the ODX-DCIS panel. Bar plots of non-Progressor vs Progressor DCIS are shown with their respective Mann-Whitney U-test results.

**Figure S8: Frequency plots of DNA Copy Number Variation (CNV) across the genome**. CNV patterns are shown across all chromosomes for each PAM50 subtype for DCIS non-progressors (Basal: n=6, Her2: n=9, LumA: n=20, LumB: n=11); DCIS progressors (Basal: n=7, Her2: n=15, LumA: n=14, LumB: n=13); and TCGA-IBC (Basal: n=186, Her2: n=91, LumA: n=552, LumB: n=208).

Genomic position is indicated on the x-axis, with p-arm on the left, q-arm on the right of each plot. The y-axis shows the frequency of deletions (below baseline, 0) or amplifications (above baseline, 0). Note differing y-axis scales, adjusted to the largest % of deletions or amplifications for each plot.

**Figure S9:** **Associations between splice complexity (s) and clinicopathologic and genomic variables**. Boxplots illustrate the median and the first and third quartiles (box); the whiskers denote range of values. **A.** Boxplots of average s-scores in DCIS progressors and non-progressors**.** ANOVA: F-statistic = 0.17, df = 1, p = 0.68. **B.** Boxplots of average DCIS sample s-scores in each PAM50 subtype: Basal; HER2; LumA: Luminal A; LumB: Luminal B; Nl-like: Normal-like. ANOVA: F-statistic = 0.35, df = 4, p = 0.26. **C.** Boxplots of average DCIS sample s-scores among expression clusters. ANOVA: F-statistic = 32.19, df = 1, p = 9.73e-08. **D.** Boxplots of average DCIS sample s-scores among methylation clusters. ANOVA: F-statistic = 1.12, df = 4, p-value = 0.35. **E.** Scatterplot of average DCIS sample s-scores by age.

**Figure S10:** **Association between splice complexity and sample read counts**.

**A.** Scatter plot of total read count vs complexity (s). Dotted line indicates mean read count. **B.** Histogram of Spearman correlation between complexity (s) and gene expression. **C.** Heatmap showing complexity (s) for most variable intron groups, samples ordered by decreasing complexity (yellow -> red). Bars across the top show PAM50 and Progression status. **D.** Heatmap showing overall expression of the same genes as in panel C. Bars across the top show PAM50 and Progression status, in the same order as panel C.
