# Supplementary material for: Multimodal genome-wide survey of progressing and non-progressing breast ductal carcinoma in-situ
Source: Breast Cancer Res. 2024 Dec 4;26:178. doi: 10.1186/s13058-024-01927-1 (PMC11616160; doi:10.1186/s13058-024-01927-1)
Supplement: Supplementary file 6 — Supplementary Material 6 [file 13058_2024_1927_MOESM6_ESM.pdf]

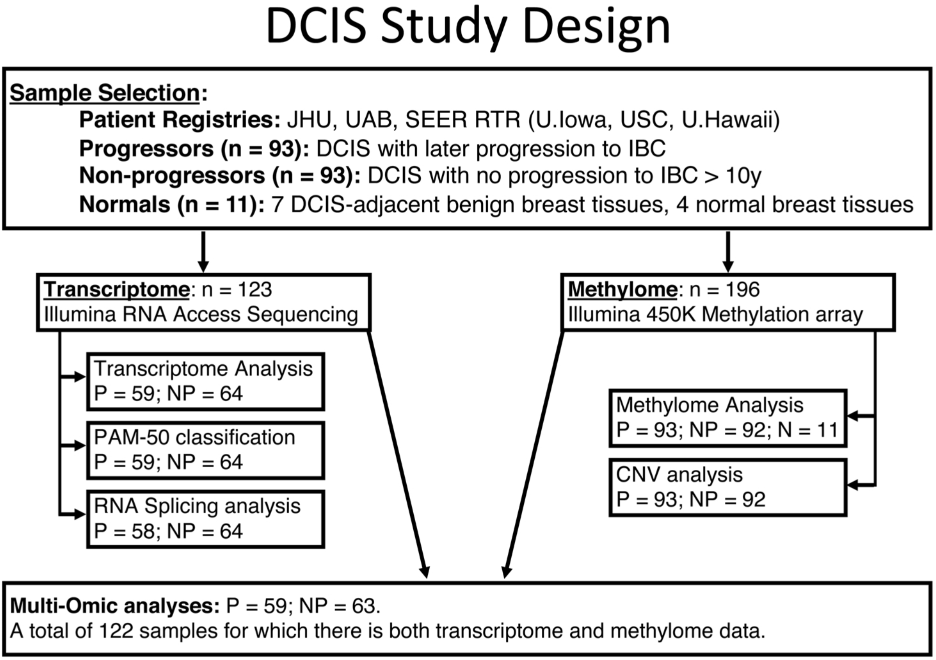
DCIS Study Design
Sample Selection:
Patient Registries: JHU, UAB, SEER RTR (U.Iowa, USC, U.Hawaii)
Progressors (n = 93): DCIS with later progression to IBC
Non-progressors (n = 93): DCIS with no progression to IBC > 10y
Normals (n = 11): 7 DCIS-adjacent benign breast tissues, 4 normal breast tissues
Transcriptome: n = 123
Illumina RNA Access Sequencing
Transcriptome Analysis
P = 59; NP = 64
PAM-50 classification
P = 59; NP = 64
RNA Splicing analysis
P = 58; NP = 64
Methylome: n = 196
Illumina 450K Methylation array
Methylome Analysis
P = 93; NP = 92; N = 11
CNV analysis
P = 93; NP = 92
Multi-Omic analyses: P = 59; NP = 63.
A total of 122 samples for which there is both transcriptome and methylome data.
