# Supplementary figures and images for: Multimodal genome-wide survey of progressing and non-progressing breast ductal carcinoma in-situ
Source: Breast Cancer Res. 2024 Dec 4;26:178. doi: 10.1186/s13058-024-01927-1 (PMC11616160; doi:10.1186/s13058-024-01927-1)

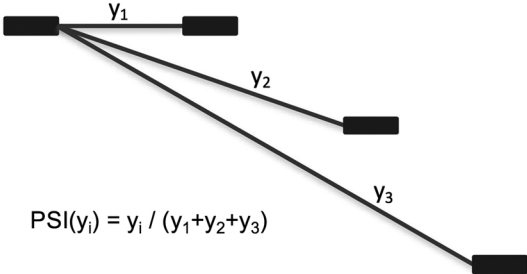

Supplement: Supplementary file 7 — Supplementary Material 7 [file 13058_2024_1927_MOESM7_ESM.pdf]

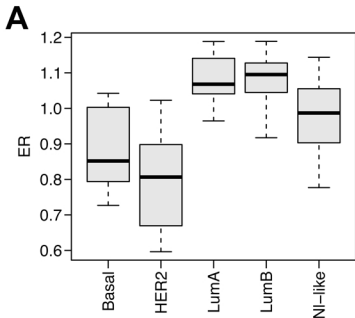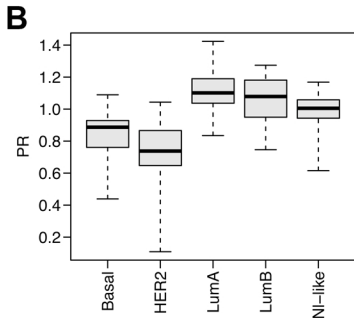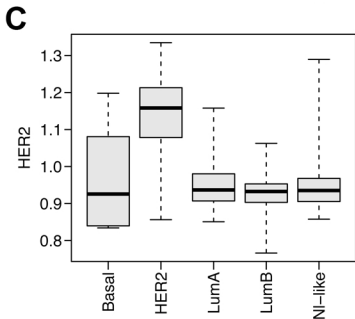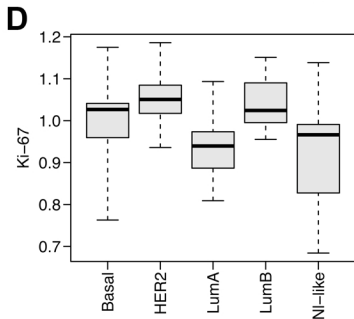

Supplement: Supplementary file 8 — Supplementary Material 8 [file 13058_2024_1927_MOESM8_ESM.pdf]

**A**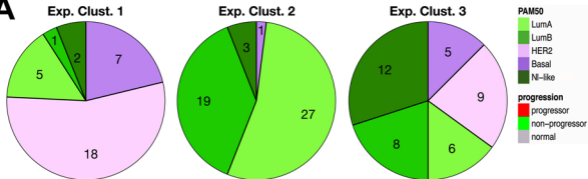**B**

Unsupervised DCIS

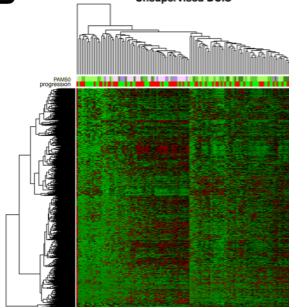**C**

TCGA

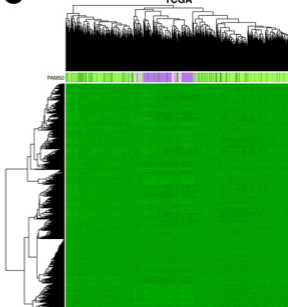

Supplement: Supplementary file 9 — Supplementary Material 9 [file 13058_2024_1927_MOESM9_ESM.pdf]

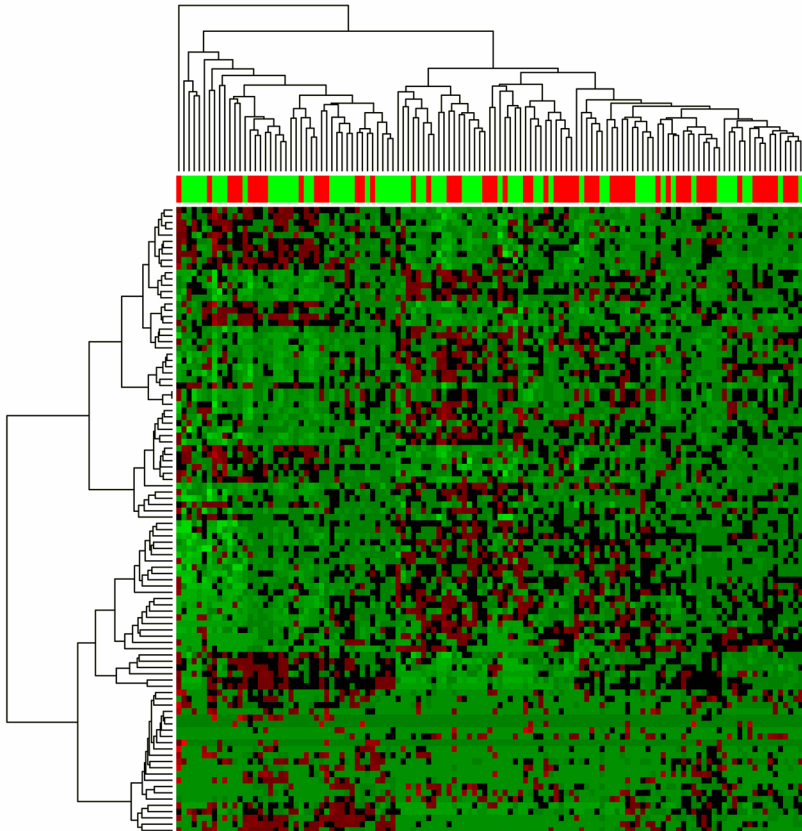

Supplement: Supplementary file 11 — Supplementary Material 11 [file 13058_2024_1927_MOESM11_ESM.pdf]

**MKI67**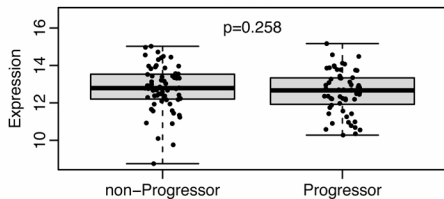**AURKA**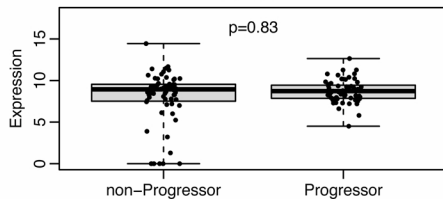**BIRC5**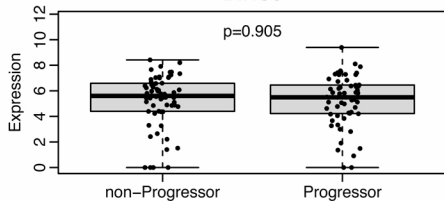**CCNB1**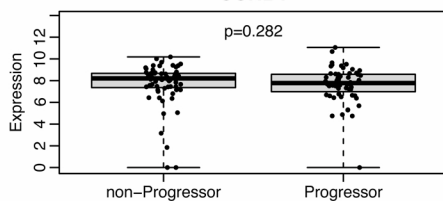**MYBL2**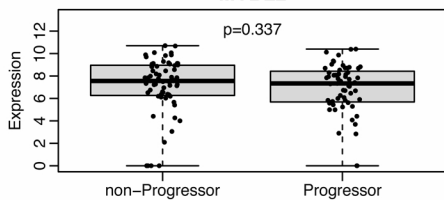

Supplement: Supplementary file 12 — Supplementary Material 12 [file 13058_2024_1927_MOESM12_ESM.pdf]

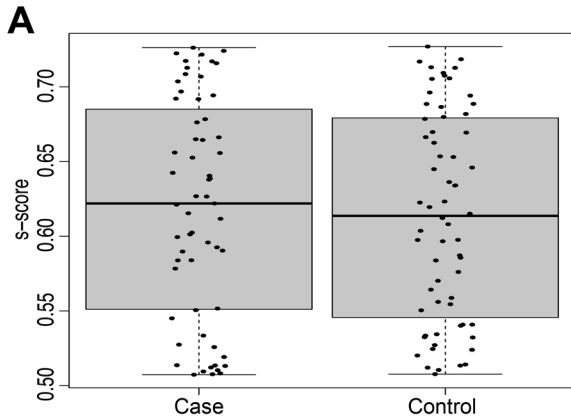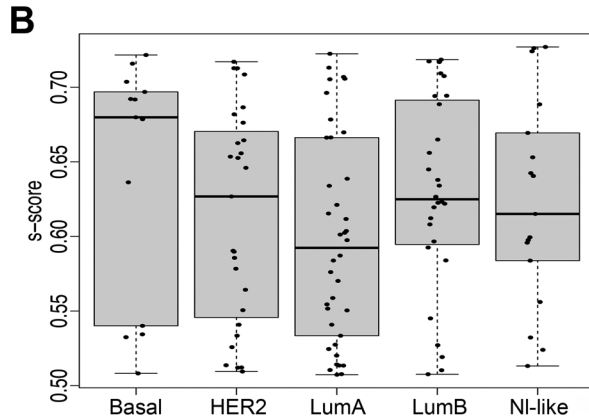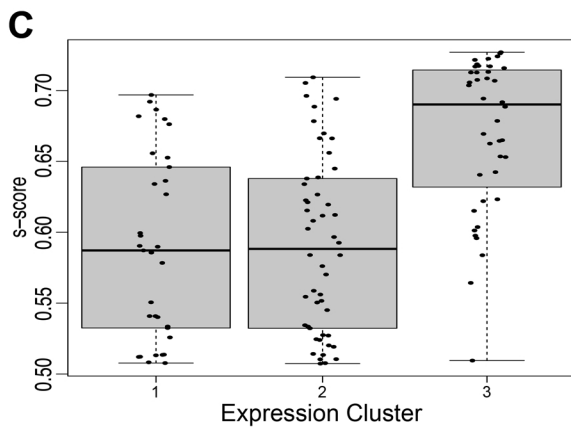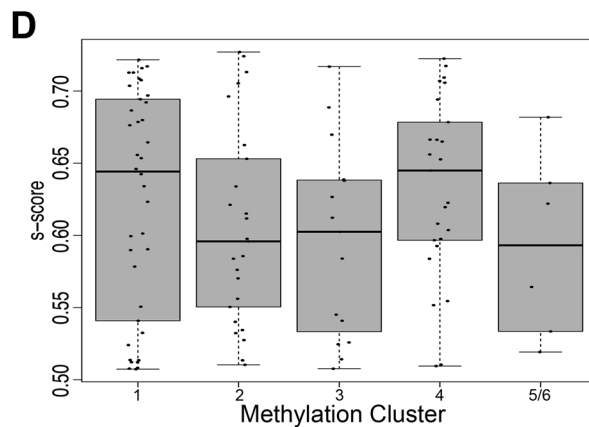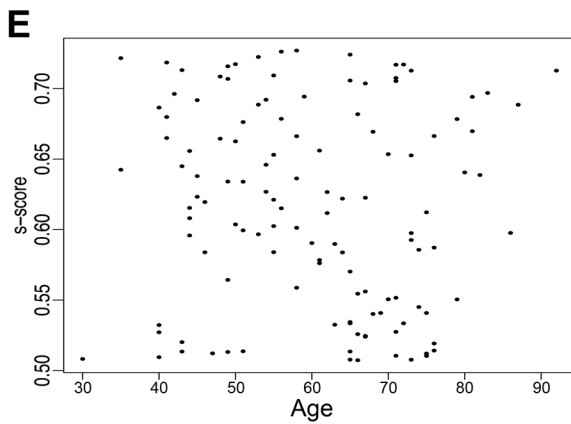

Supplement: Supplementary file 14 — Supplementary Material 14 [file 13058_2024_1927_MOESM14_ESM.pdf]

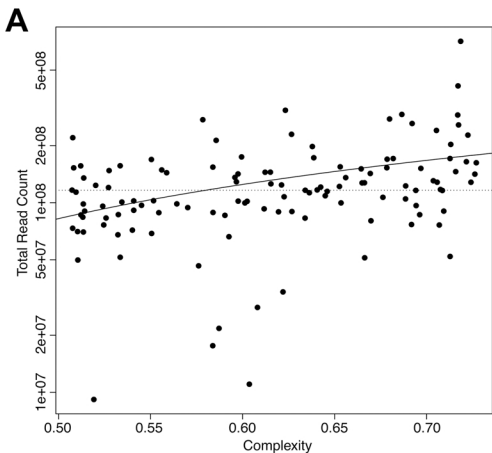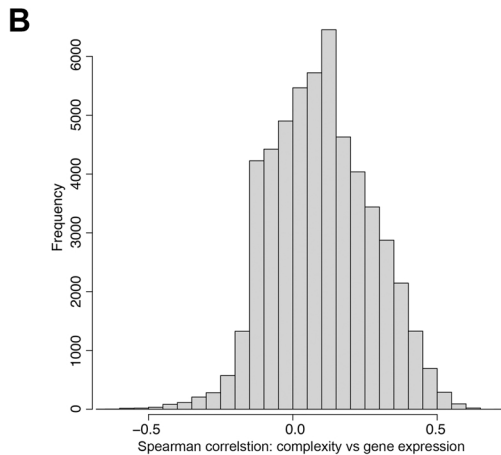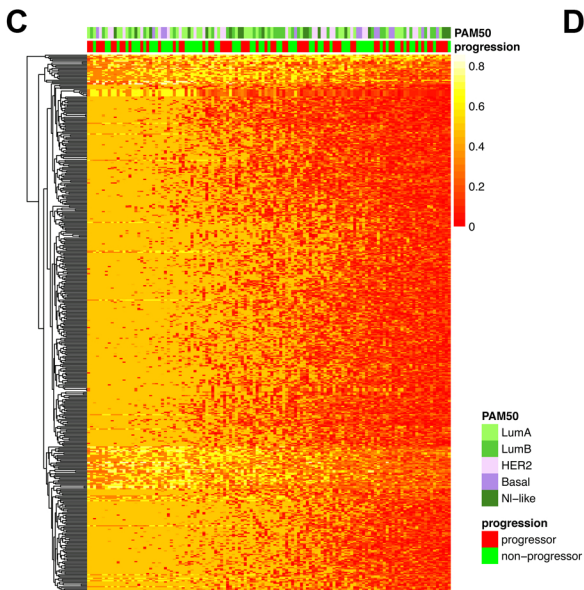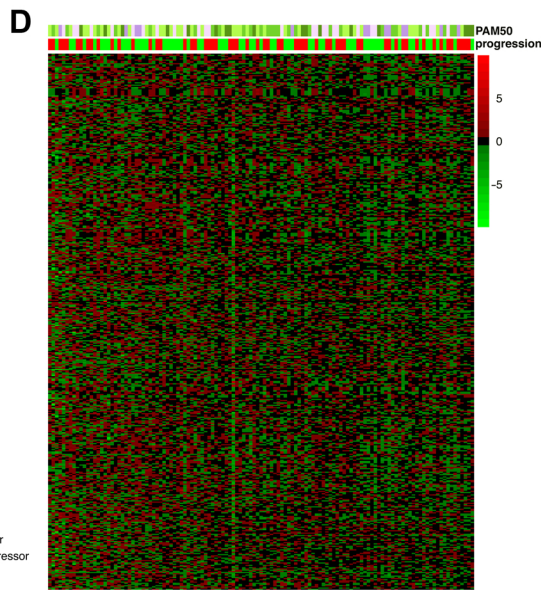

Supplement: Supplementary file 15 — Supplementary Material 15 [file 13058_2024_1927_MOESM15_ESM.pdf]
