# Supplementary material for: Multimodal genome-wide survey of progressing and non-progressing breast ductal carcinoma in-situ
Source: Breast Cancer Res. 2024 Dec 4;26:178. doi: 10.1186/s13058-024-01927-1 (PMC11616160; doi:10.1186/s13058-024-01927-1)

Figure S5 A: PAM50 vs Expression Clusters

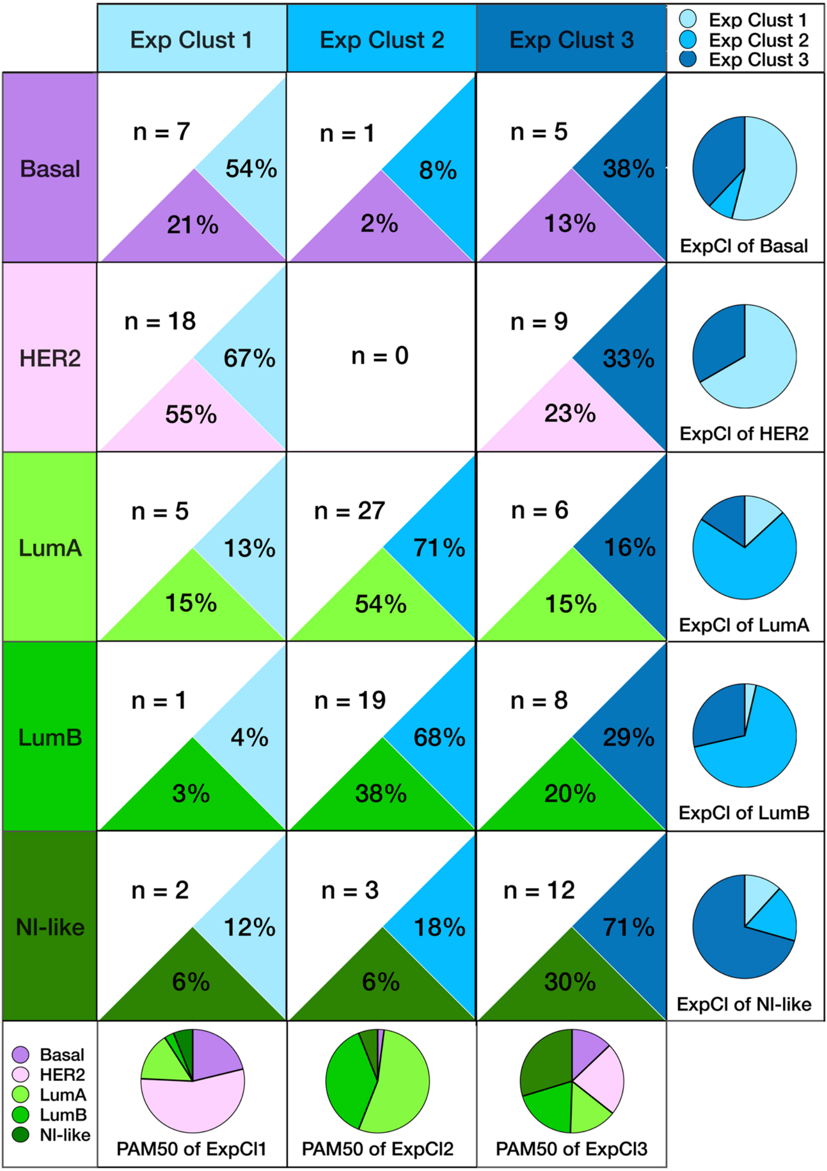

Figure S5 B: PAM50 vs. Outcomes

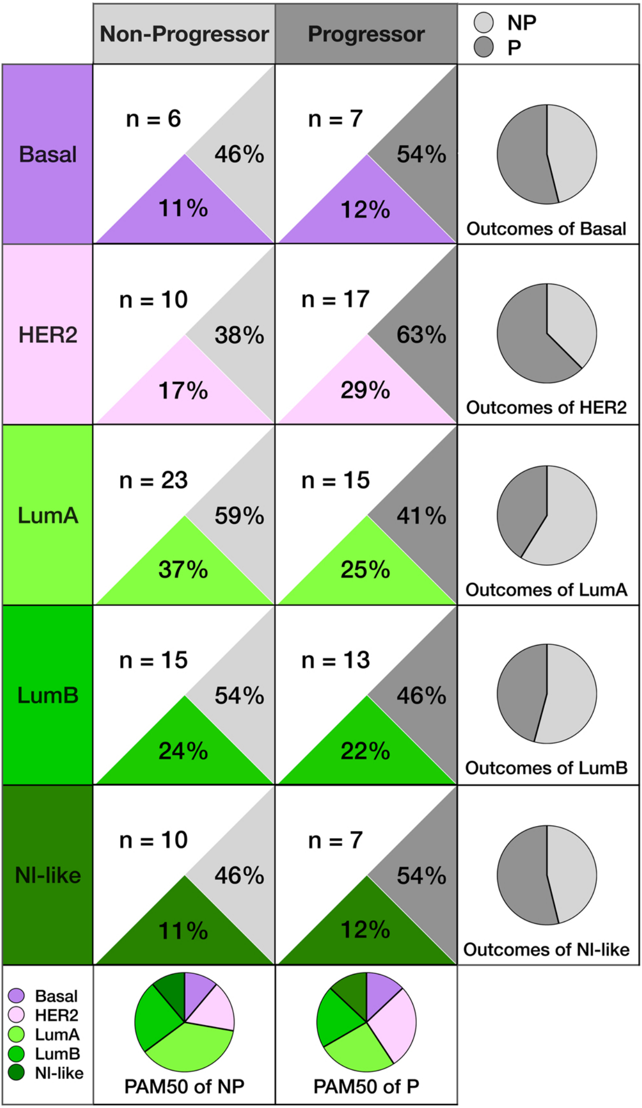

Figure S5 C: PAM50 vs Methylation Clusters

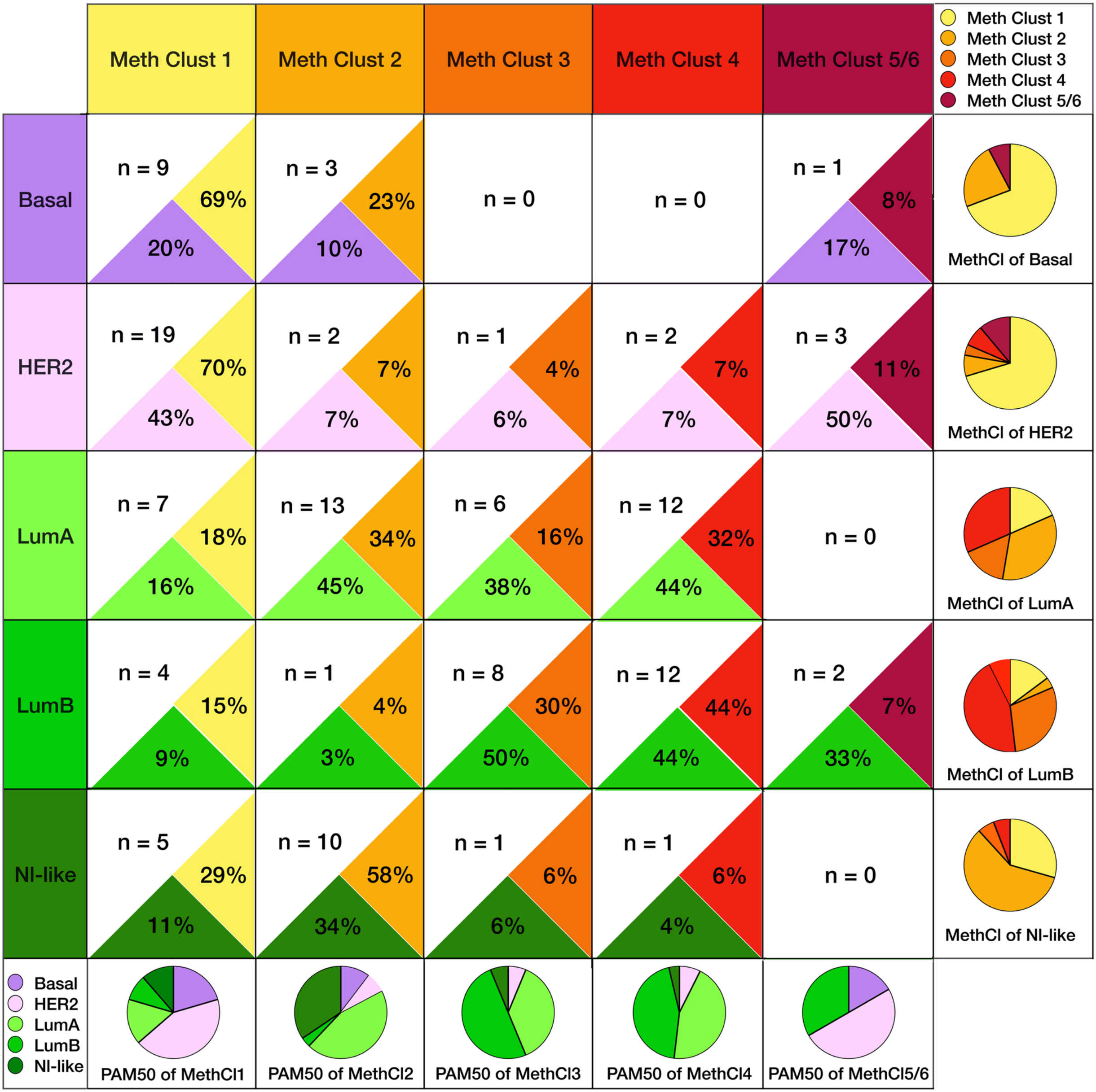

Figure S5 D: Methylation Clusters vs Expression Clusters

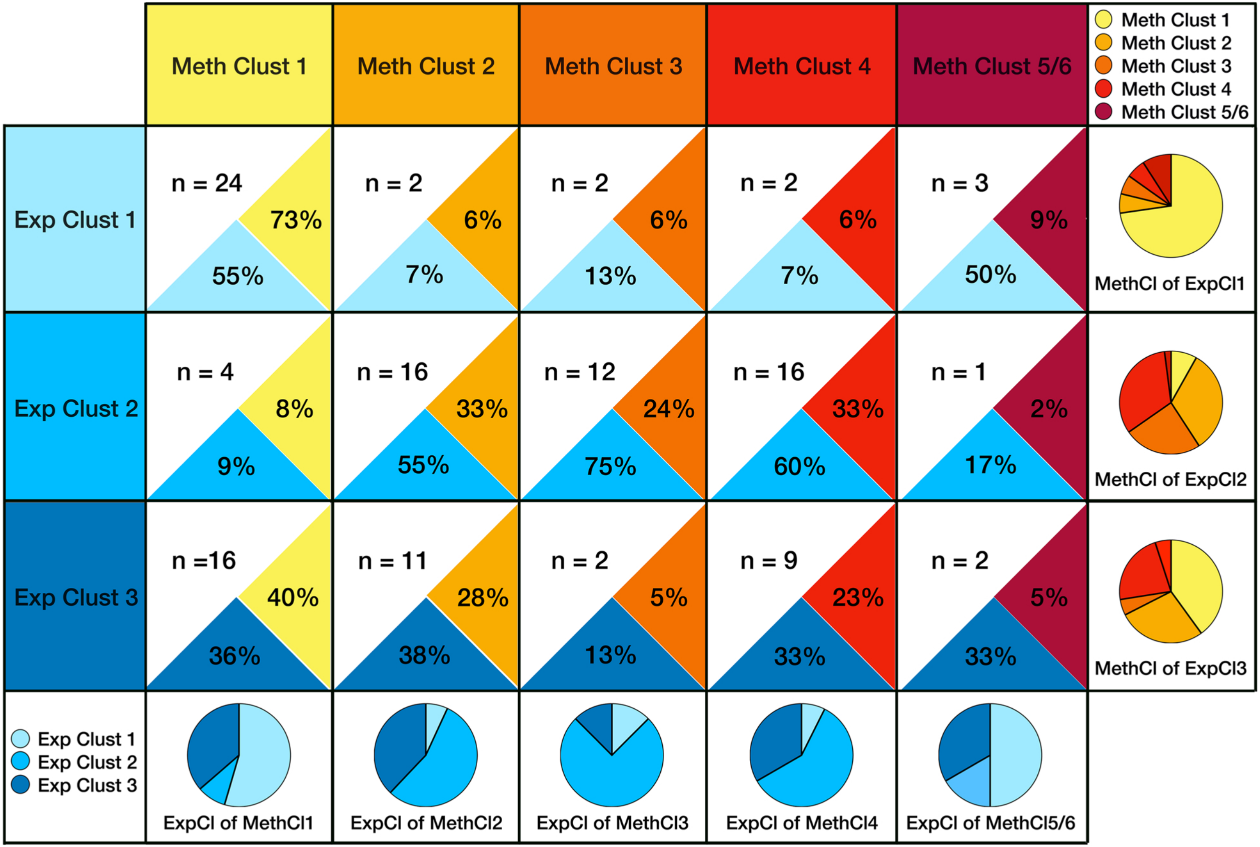

Figure S5 E: Methylation Clusters vs. Outcomes

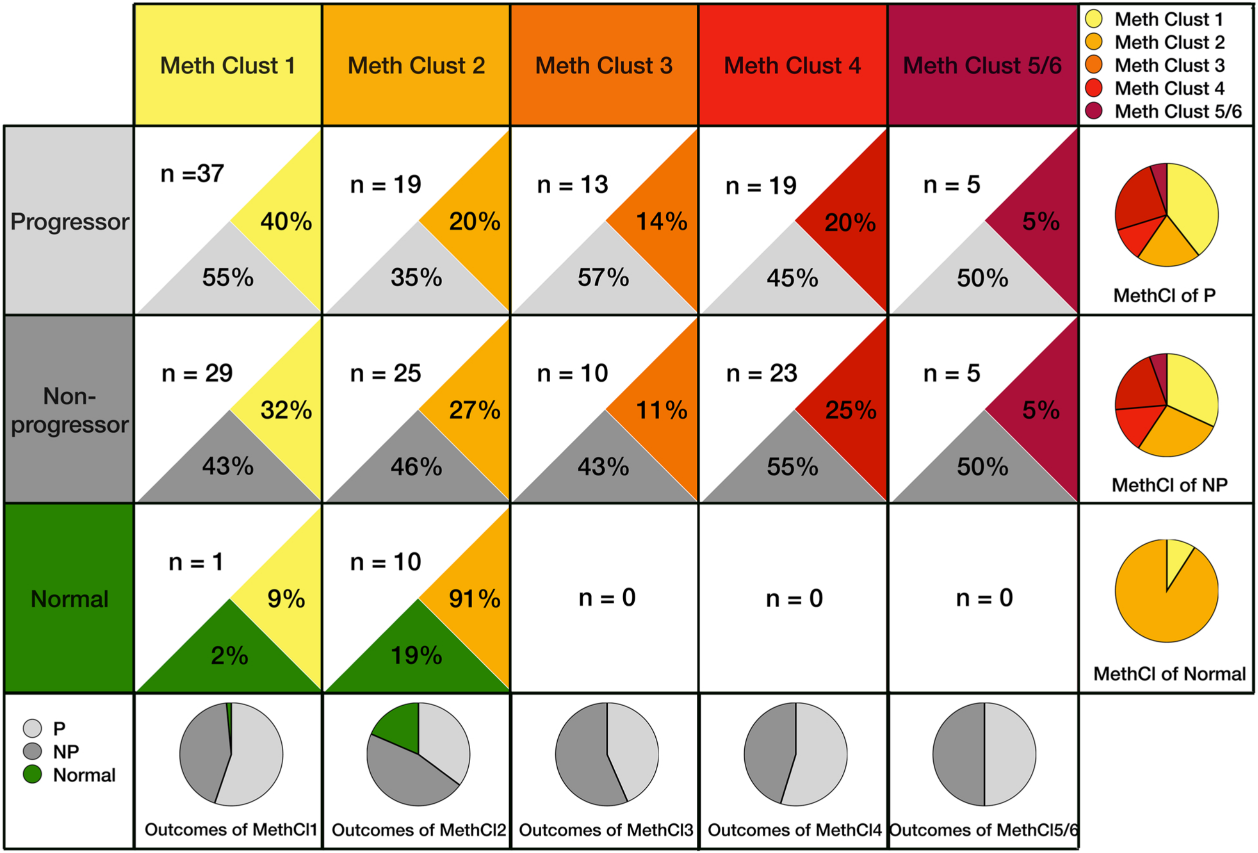

Figure S5 F: Expression Clusters vs. Outcomes

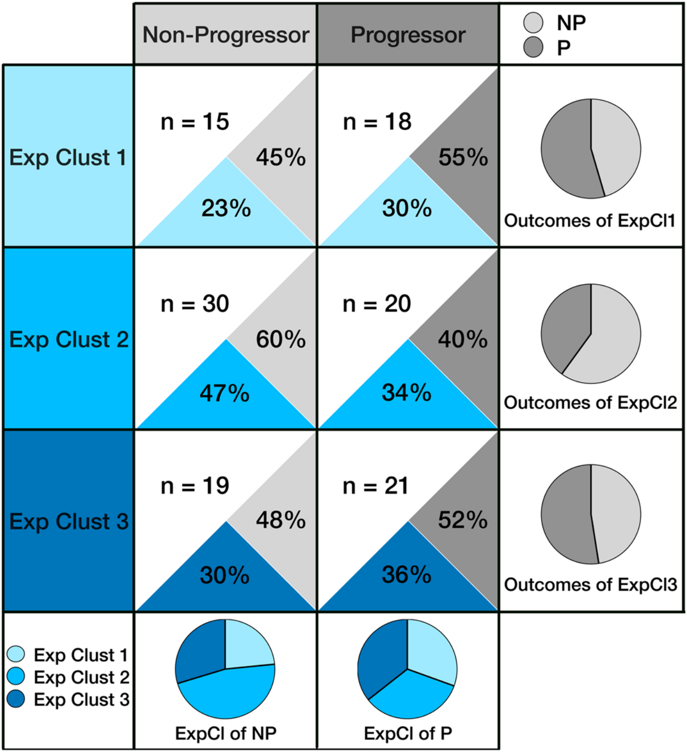

Supplement: Supplementary file 10 — Supplementary Material 10 [file 13058_2024_1927_MOESM10_ESM.pdf]
