# Supplementary material for: Multimodal genome-wide survey of progressing and non-progressing breast ductal carcinoma in-situ
Source: Breast Cancer Res. 2024 Dec 4;26:178. doi: 10.1186/s13058-024-01927-1 (PMC11616160; doi:10.1186/s13058-024-01927-1)
Supplement: Supplementary file 13 — Supplementary Material 13 [file 13058_2024_1927_MOESM13_ESM.pdf]

CHR 1

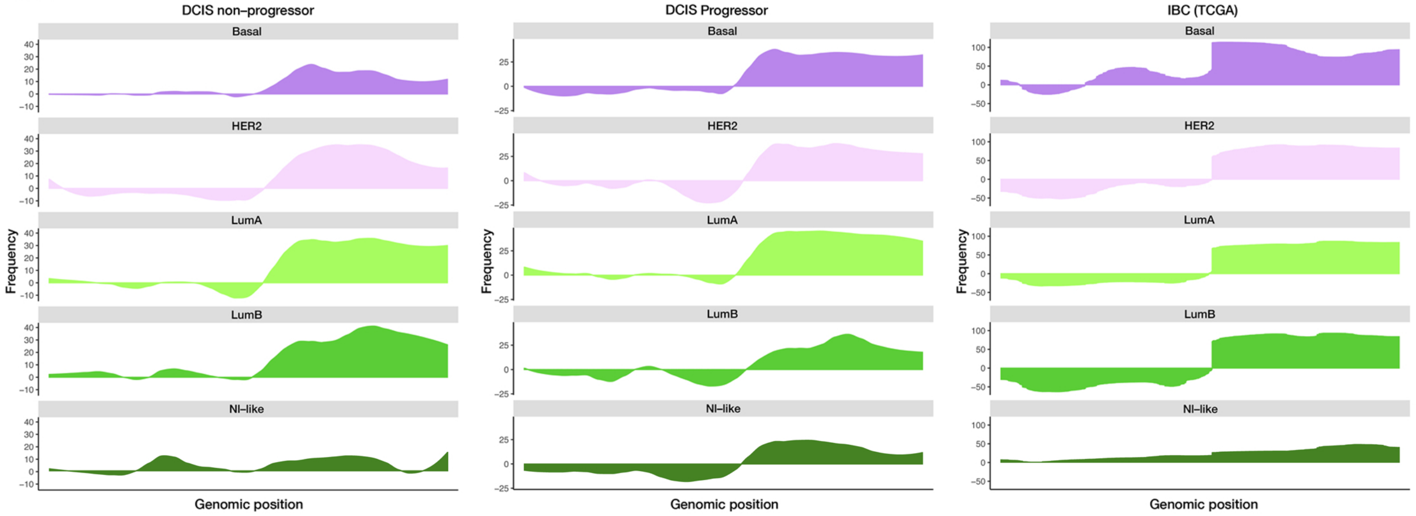

CHR 2

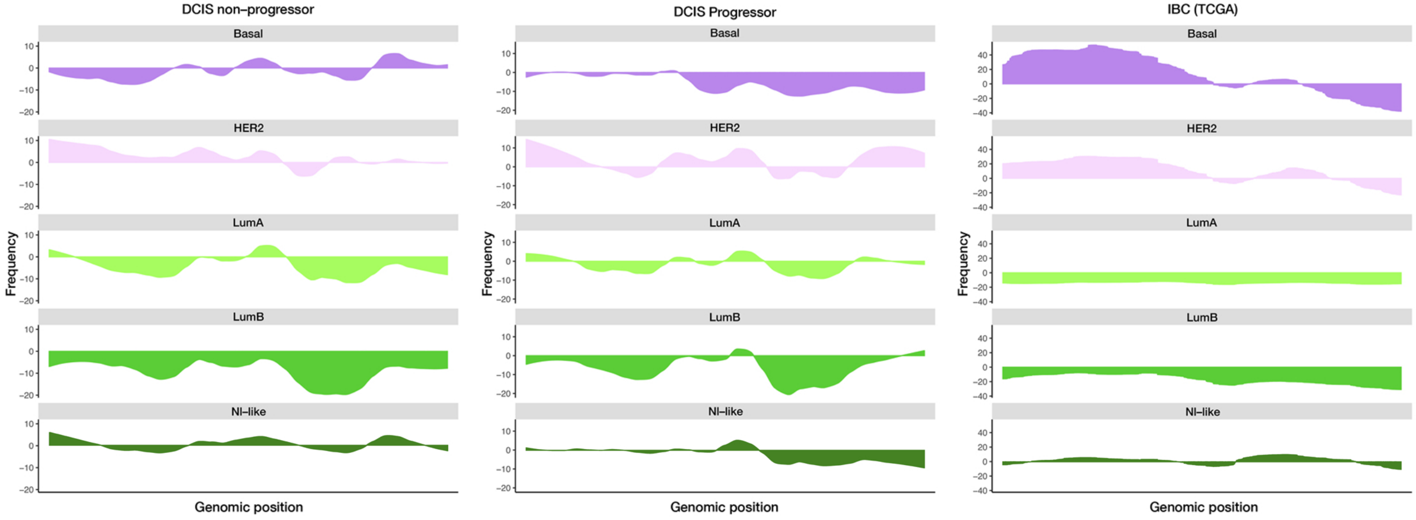

CHR 3

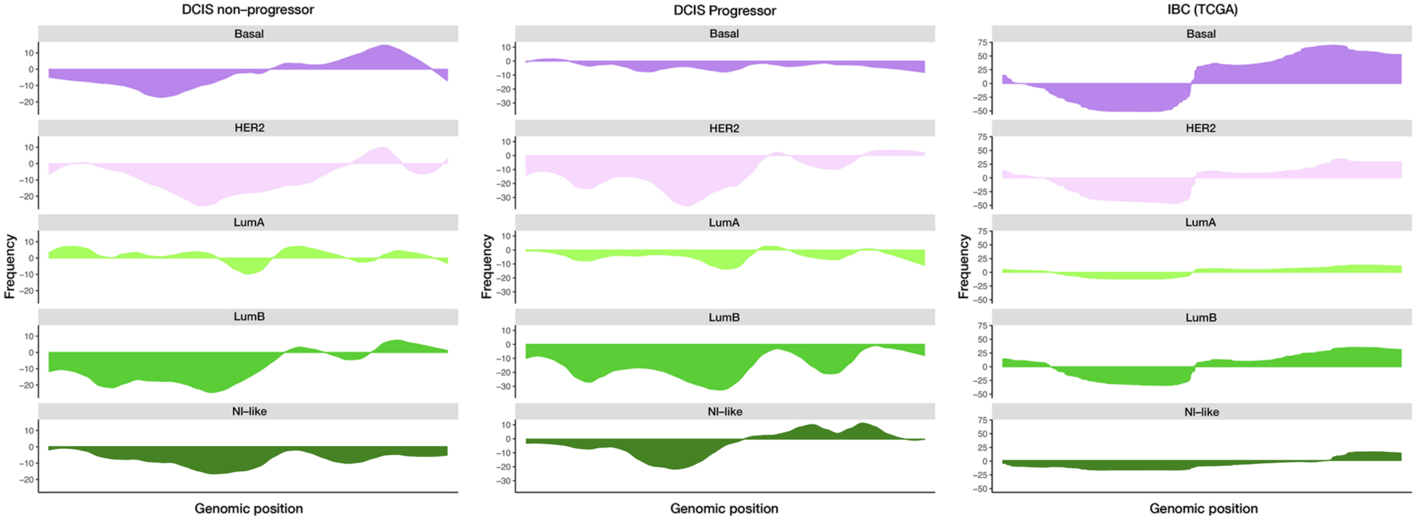

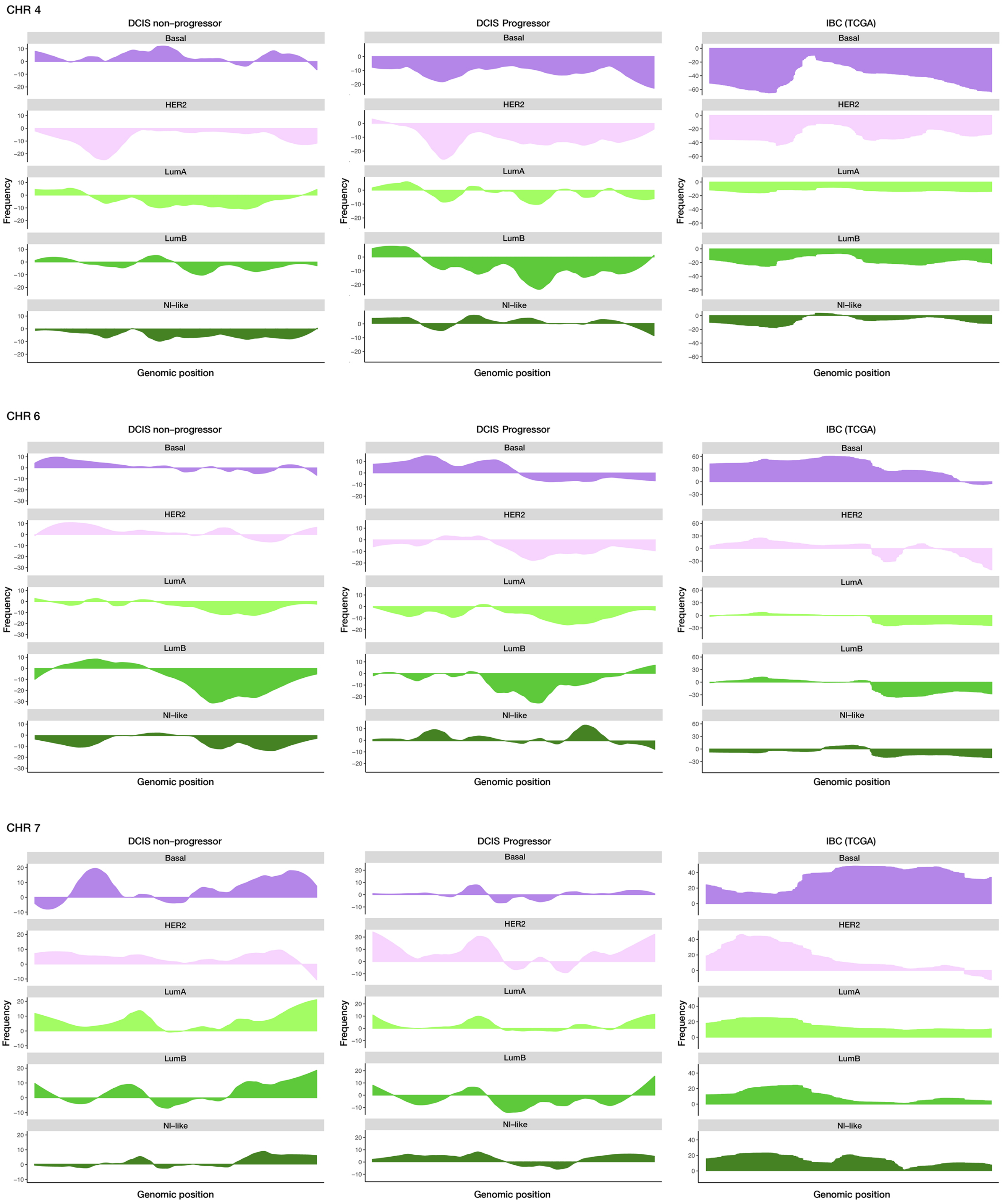

CHR 4
DCIS non–progressor
DCIS Progressor
IBC (TCGA)
Basal
HER2
LumA
LumB
Nl–like
Frequency
Genomic position
CHR 6
DCIS non–progressor
DCIS Progressor
IBC (TCGA)
Basal
HER2
LumA
LumB
Nl–like
Frequency
Genomic position
CHR 7
DCIS non–progressor
DCIS Progressor
IBC (TCGA)
Basal
HER2
LumA
LumB
Nl–like
Frequency
Genomic position

CHR 8

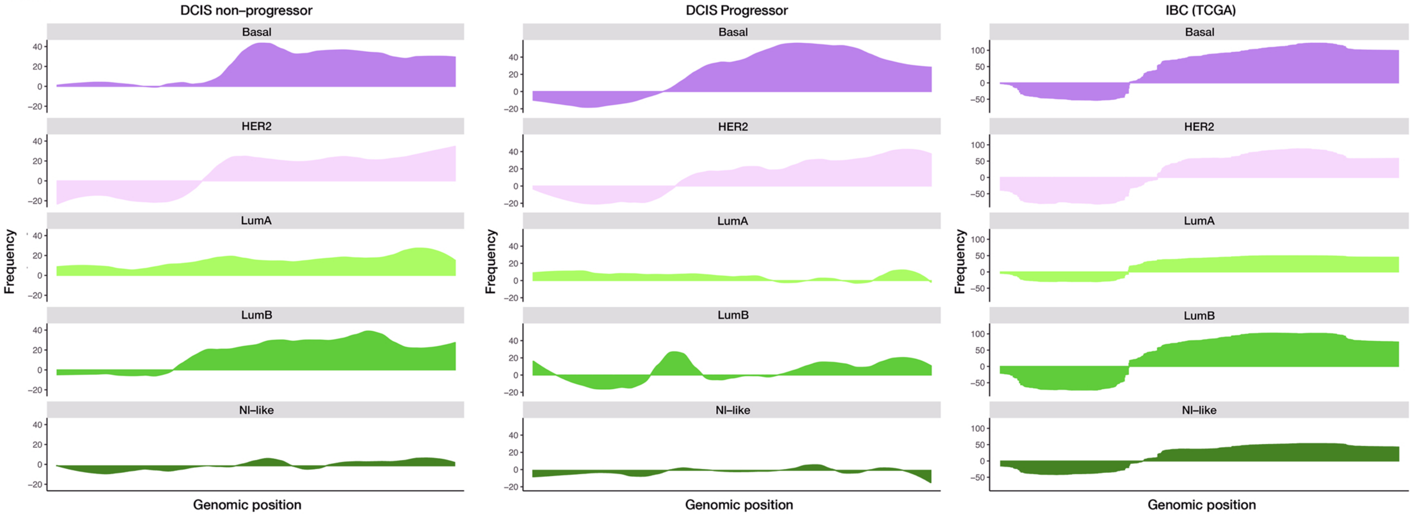

CHR 9

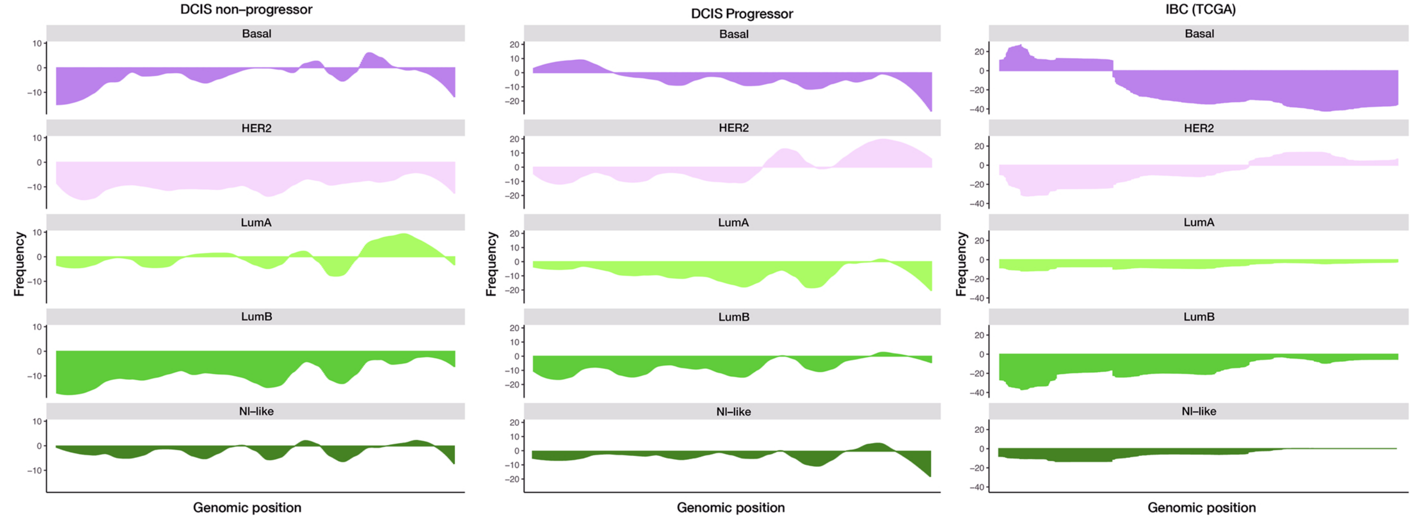

CHR 10

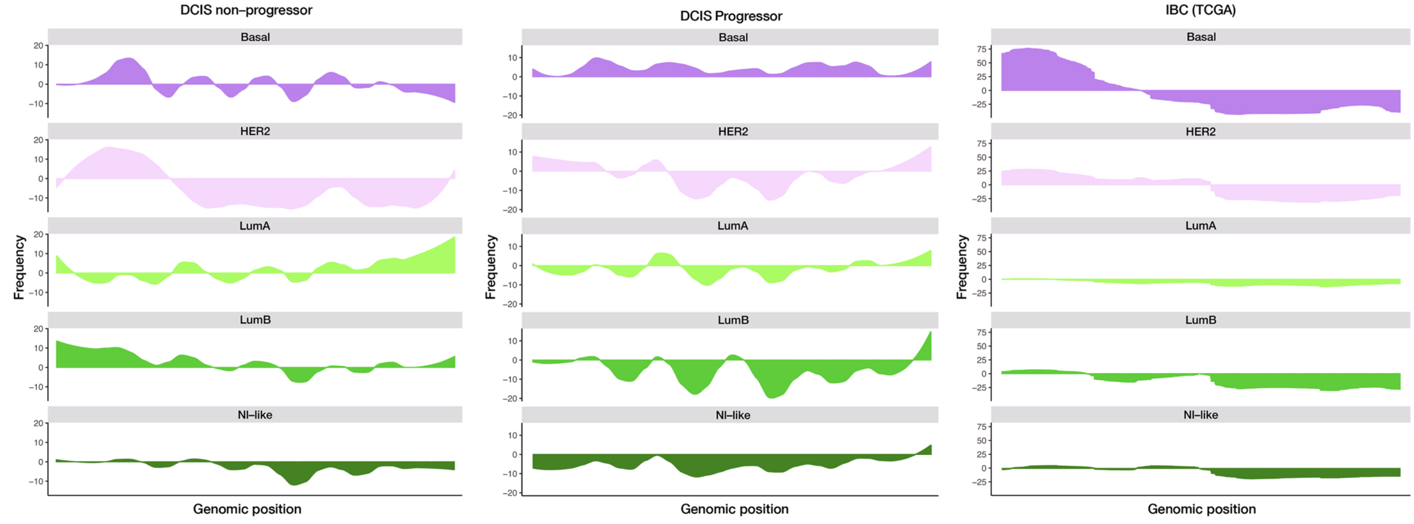

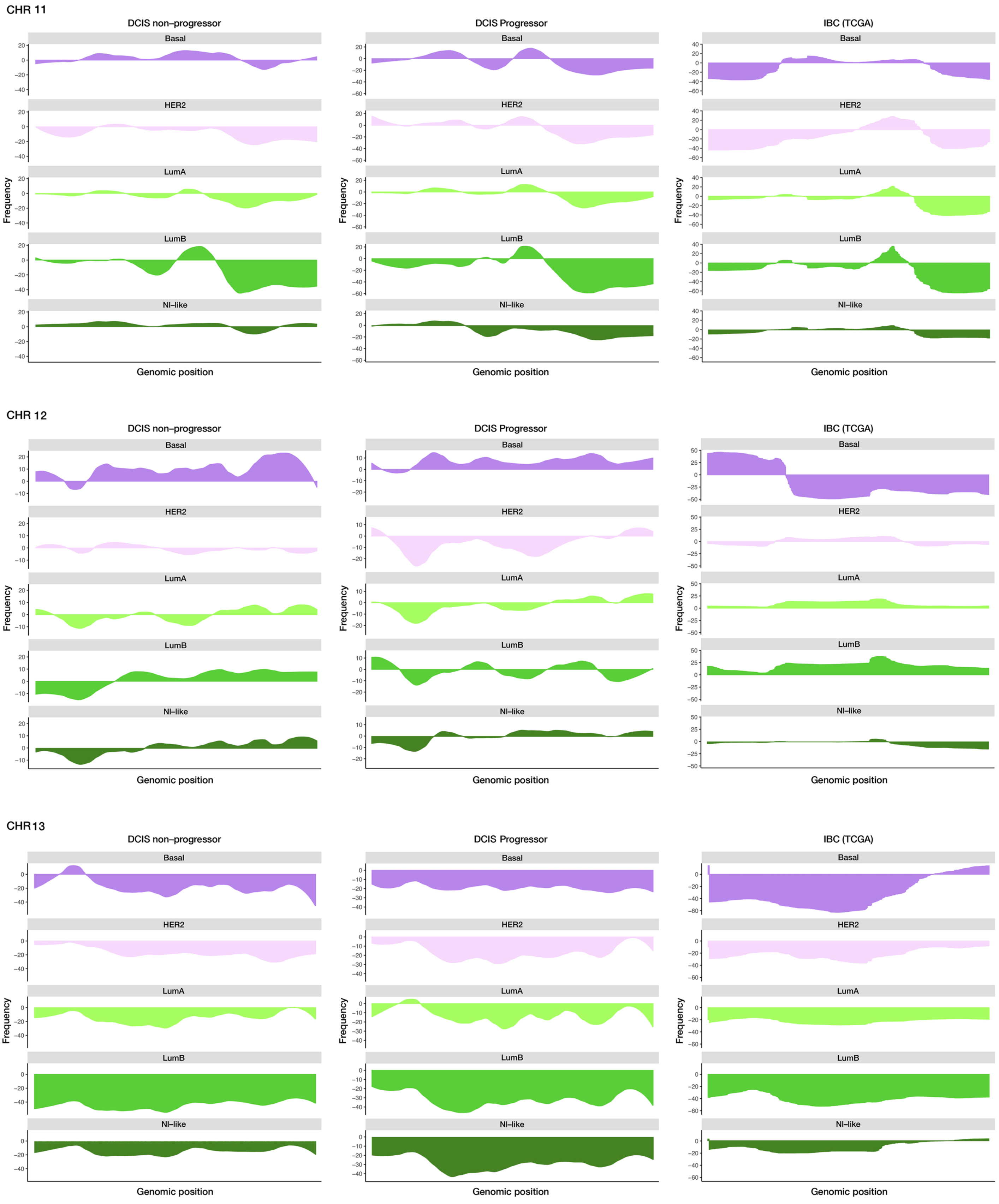
CHR 11
DCIS non–progressor
DCIS Progressor
IBC (TCGA)
Basal
HER2
LumA
LumB
Nl–like
Frequency
Genomic position
CHR 12
DCIS non–progressor
DCIS Progressor
IBC (TCGA)
Basal
HER2
LumA
LumB
Nl–like
Frequency
Genomic position
CHR 13
DCIS non–progressor
DCIS Progressor
IBC (TCGA)
Basal
HER2
LumA
LumB
Nl–like
Frequency
Genomic position

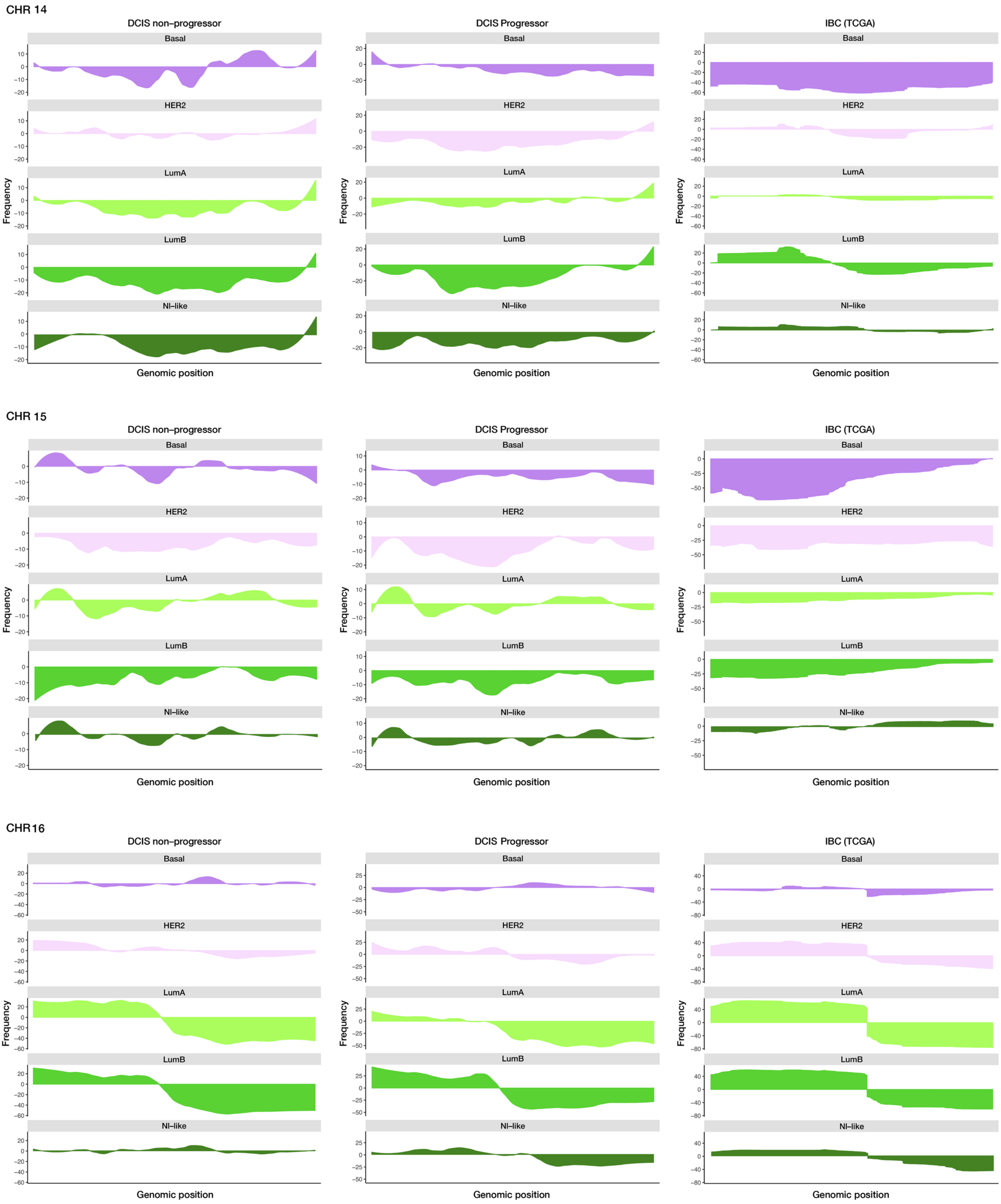
CHR 14
DCIS non–progressor
DCIS Progressor
IBC (TCGA)
Basal
HER2
LumA
LumB
Nl–like
Frequency
Genomic position
CHR 15
DCIS non–progressor
DCIS Progressor
IBC (TCGA)
Basal
HER2
LumA
LumB
Nl–like
Frequency
Genomic position
CHR 16
DCIS non–progressor
DCIS Progressor
IBC (TCGA)
Basal
HER2
LumA
LumB
Nl–like
Frequency
Genomic position

CHR 17

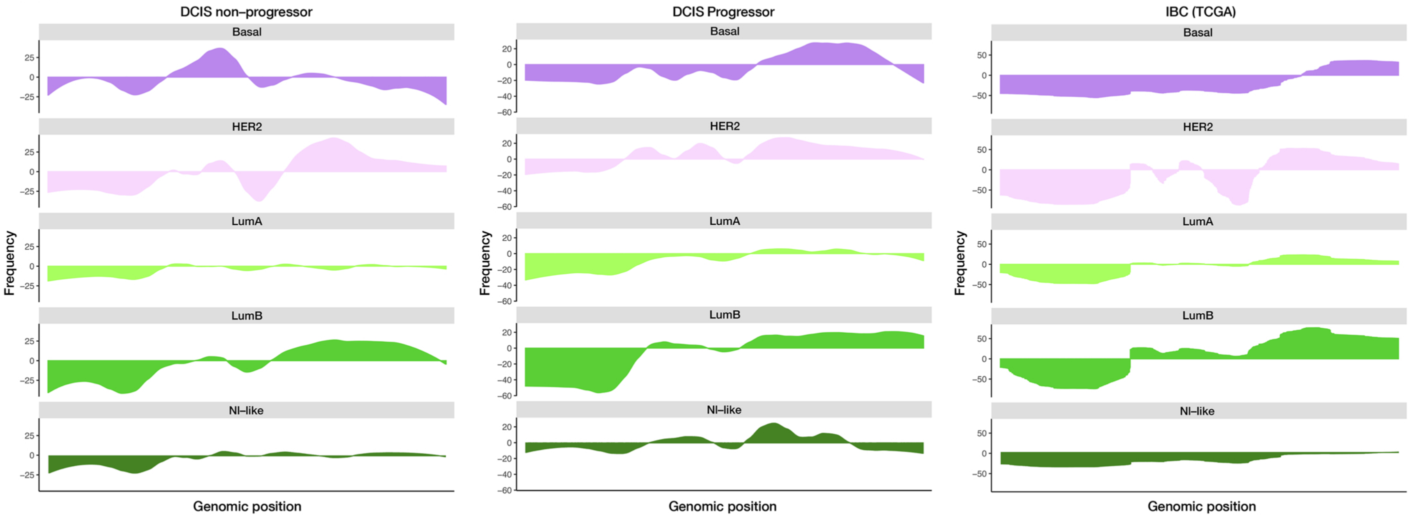

CHR 18

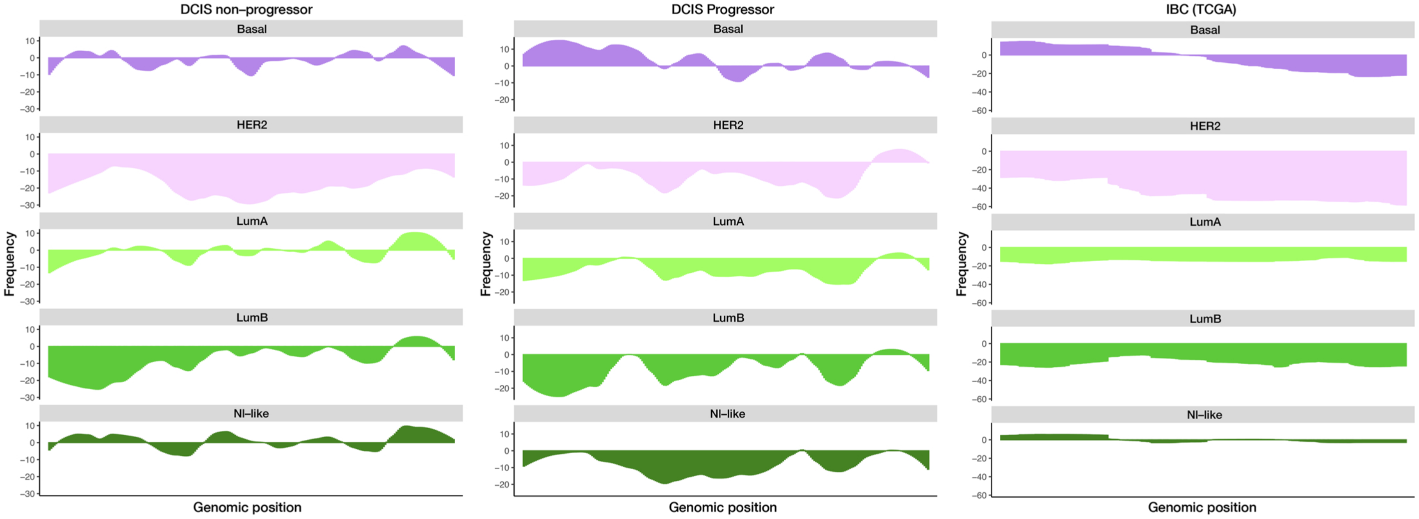

CHR 19

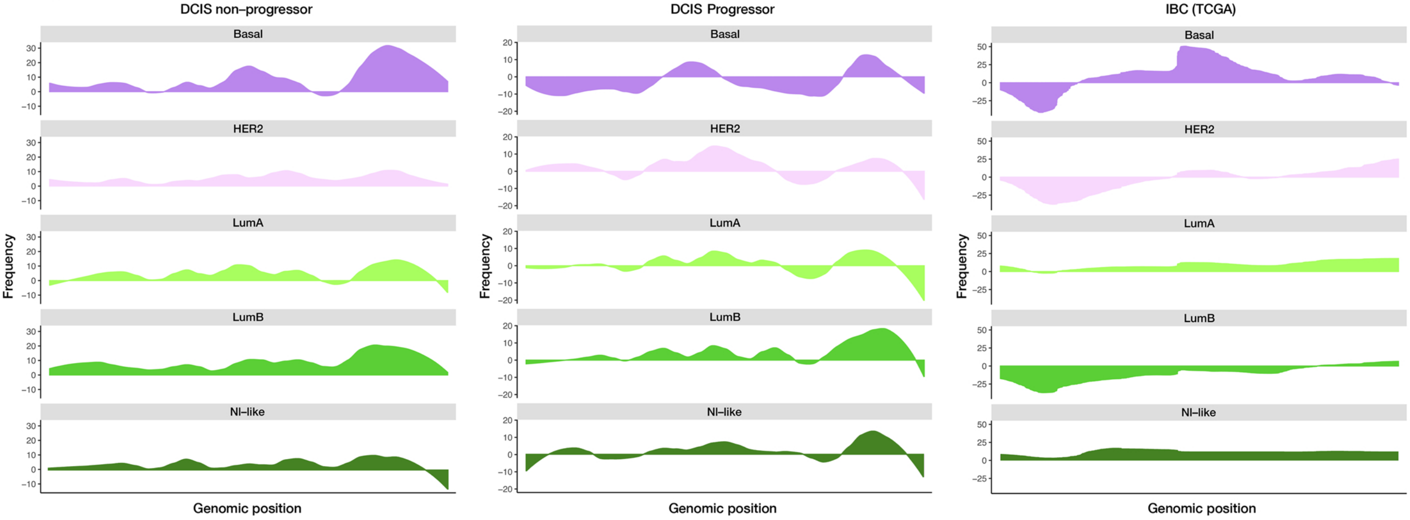

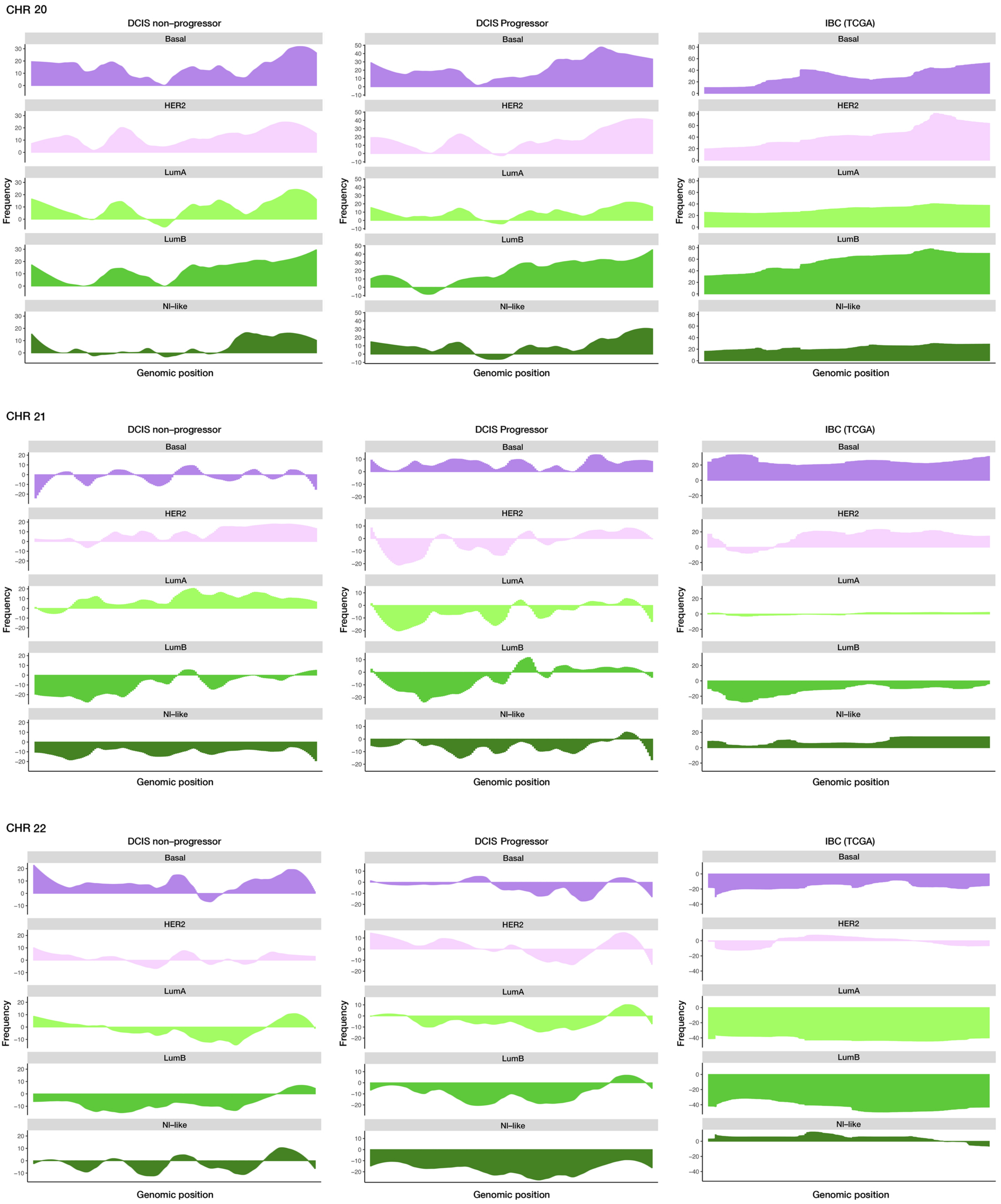
CHR 20
DCIS non–progressor
DCIS Progressor
IBC (TCGA)
Basal
HER2
LumA
LumB
Nl–like
Frequency
Genomic position
CHR 21
DCIS non–progressor
DCIS Progressor
IBC (TCGA)
Basal
HER2
LumA
LumB
Nl–like
Frequency
Genomic position
CHR 22
DCIS non–progressor
DCIS Progressor
IBC (TCGA)
Basal
HER2
LumA
LumB
Nl–like
Frequency
Genomic position
